# Enzymatic separation of epimeric 4-*C*-hydroxymethylated furanosugars: Synthesis of bicyclic nucleosides

**DOI:** 10.3762/bjoc.13.205

**Published:** 2017-10-05

**Authors:** Neha Rana, Manish Kumar, Vinod Khatri, Jyotirmoy Maity, Ashok K Prasad

**Affiliations:** 1 Bioorganic Laboratory, Department of Chemistry, University of Delhi, Delhi-110 007, India; Phone: 00-91-11-27662486

**Keywords:** bicyclonucleosides, biocatalysis, lipase, Novozyme^®^-435, separation of epimers

## Abstract

Conversion of D-glucose to 4-*C-*hydroxymethyl-1,2-*O*-isopropylidene-α-D-ribofuranose, which is a key precursor for the synthesis of different types of bicyclic/spiro nucleosides, led to the formation of an inseparable 1:1 mixture of the desired product and 4-*C-*hydroxymethyl-1,2-*O*-isopropylidene-α-D-xylofuranose. A convenient environment friendly Novozyme^®^-435 catalyzed selective acetylation methodology has been developed for the separation of an epimeric mixture of ribo*-* and xylotrihydroxyfuranosides in quantitative yields. The structure of both the monoacetylated epimers, i.e., 5-*O*-acetyl-4-*C*-hydroxymethyl-1,2-*O*-isopropylidene-α-D-ribo- and xylofuranose obtained by enzymatic acetylation, has been confirmed by an X-ray study on their corresponding 4-*C*-*p*-toluenesulfonyloxymethyl derivatives. Furthermore, the two separated epimers were used for the convergent synthesis of two different types of bicyclic nucleosides, which confirms their synthetic utility.

## Introduction

Sugar-modified bicyclic nucleosides have drawn the attention of synthetic chemists because of their effect on the conformational restriction of the furanose moiety of the nucleoside [1–9]. The conformational restriction has led to the enhancement in target selectivity and in vivo stability of the nucleoside-based drug candidates. One of the important precursors for the synthesis of different types of bicyclonucleosides is 4-*C*-hydroxymethyl-1,2-*O*-isopropylidene-α-D-ribofuranose. The synthesis of the ribo*-*trihydroxy sugar derivative starting from diacetone-D-glucose led to the formation of an inseparable 1:1 mixture of the required compound and its C-3 epimer, i.e., 4-*C-*hydroxymethyl-1,2-*O*-isopropylidene-α-D-xylofuranose [10].

Lipases have been used extensively for the selective manipulation of hydroxy groups present in different sugars and sugar moieties of synthetic or naturally occurring glycosides, nucleosides, etc. Gotor et al. [11] have reported a lipase-mediated acylation of an equimolecular mixture of D/L-thymidine with acetonoxime levulinate as acylating agent and *Pseudomonas cepacia* lipase as biocatalyst. Similar applications of lipases have been reported for the separation of mixtures of arabinofuranosyl and -pyranosyl nucleosides [12], *O*-aryl α,β-D-ribofuranosides, etc. [13–15]. We herein report for the first time the use of Novozyme^®^-435 for the separation of an epimeric mixture of xylo*-* and ribofuranosides. Separated epimers were further used as sugar precursors for the convergent synthesis of two different types of bicyclic nucleosides which are monomers of oxetano- and locked nucleic acids of medicinal importance [16].

## Results and Discussion

**Scheme 1:**
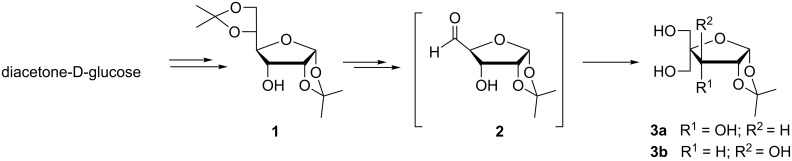
Formation of a 1:1 epimeric mixture of **3a** and **3b**.

4-*C*-Hydroxymethyl-1,2-*O*-isopropylidene-α-D-ribofuranose (**3a**) can be obtained from D-glucose via diacetonylation followed by selective deprotection of 5,6-isopropylidene protection, sodium periodate oxidation of the vicinal diol and mixed aldol–Cannizaro reaction on the resulted aldehyde **2**. However, this methodology always leads to the formation of an inseparable 1:1 mixture of 4-*C*-hydroxymethyl-1,2-*O*-isopropylidene-α-D-ribo*/*xylofuranose (**3a**,**b**, Scheme 1) [10].

**Scheme 2:**
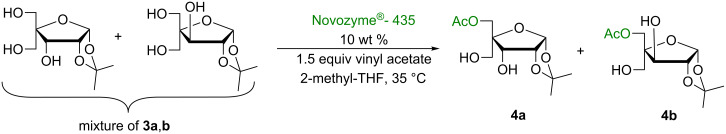
Lipase-catalysed separation of a mixture of ribo*-* and xylotrihydroxyfuranosides.

**Figure 1:**
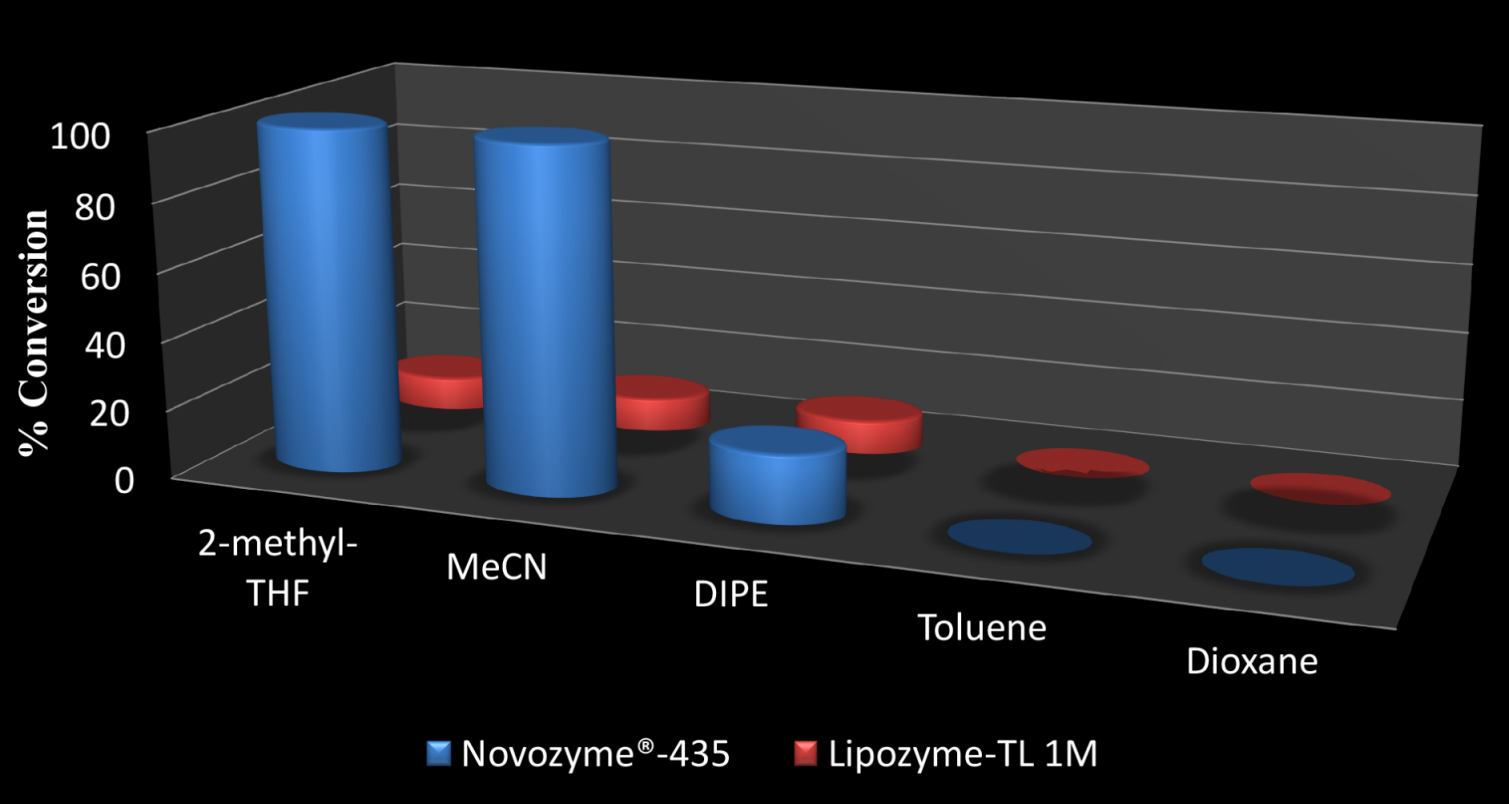
Screening of Novozyme^®^-435 and Lipozyme TL IM in different organic solvents at 35 °C for regioselective acetylation of trihydroxyribo*/*xylofuranose **3a** and **3b** (none of the reactions yielded any product when performed in the absence of the enzyme).

Separation of the mixture of 4-*C*-hydroxymethyl-1,2-*O*-isopropylidene-α-D-ribo*/*xylofuranose (**3a**,**b**) has been achieved using a selective lipase catalyzed reaction. We screened two different lipases, i.e., *Thermomyces lanuginosus* lipase immobilized on silica (Lipozyme^®^ TL IM) and *Candida antarctica* lipase-B immobilized on polyacrylate (Lewatit), commonly known as Novozyme^®^-435 in five different organic solvents, viz. 2-methyltetrahydofuran (2-methyl-THF), acetonitrile (MeCN), diisopropyl ether (DIPE), toluene and dioxane. We carried out all ten sets of reactions for diastereoselective acetylation of epimeric mixtures of ribo- and xylotrihydroxyfuranosides **3a**,**b** by using vinyl acetate at 30, 35, 40 and 45 °C and at 250 rpm in an incubator shaker to evaluate the appropriate lipase and the reaction conditions. Among the two screened lipases, Novozyme^®^-435 (10% w/w of the substrate) in 2-methyl-THF and MeCN at 35 °C was found to exhibit exclusive selectivity for the transfer of the acetyl group from vinyl acetate to the C-5 position of 4-*C*-hydroxymethyl-1,2-*O*-isopropylidene-α-D-ribofuranose (**3a**) and 4-*C*-hydroxymethyl-1,2-*O*-isopropylidene-α-D-xylofuranose (**3b**) to afford 5-*O-*acetyl-4-*C*-hydroxymethyl-1,2-*O*-isopropylidene-α-D-ribofuranose (**4a**) and 5-*O-*acetyl-4-*C*-hydroxymethyl-1,2-*O*-isopropylidene-α-D-xylofuranose (**4b**) in quantitative yields, respectively (Scheme 2 and Figure 1). Out of the two suitable solvents identified from the screening test, 2-methyl-THF as an environmentally benign solvent was used for further enzymatic separation reactions.

In a classical enzymatic reaction, a mixture of ribo*-* and xylotrihydroxyfuranosides **3a**,**b** was incubated with Novozyme^®^-435 in 2-methyl-THF using vinyl acetate as acetyl donor in an incubator shaker at 250 rpm and at 35 °C. We followed the progress of the reaction by using analytical TLC. On complete conversion of the starting materials into the corresponding products, the enzyme was filtered off to quench the reaction and the filtrate was concentrated under reduced pressure to afford a colourless oil. The two products formed in the reaction had different polarity and were easily separated by column chromatography over silica gel in quantitative yields. The structure elucidation of the two products revealed that Novozyme^®^-435 exhibited exclusive selectivity for the acetylation of C-5 hydroxy group of the substrate **3a**,**b** to afford 5-*O-*acetylated ribo*-* and xylofuranose derivatives **4a** and **4b**, respectively, in 42% and 46% yields, calculated on the basis of individual share of the trihydroxy furanoses in the mixture (Scheme 2).

**Figure 2:**
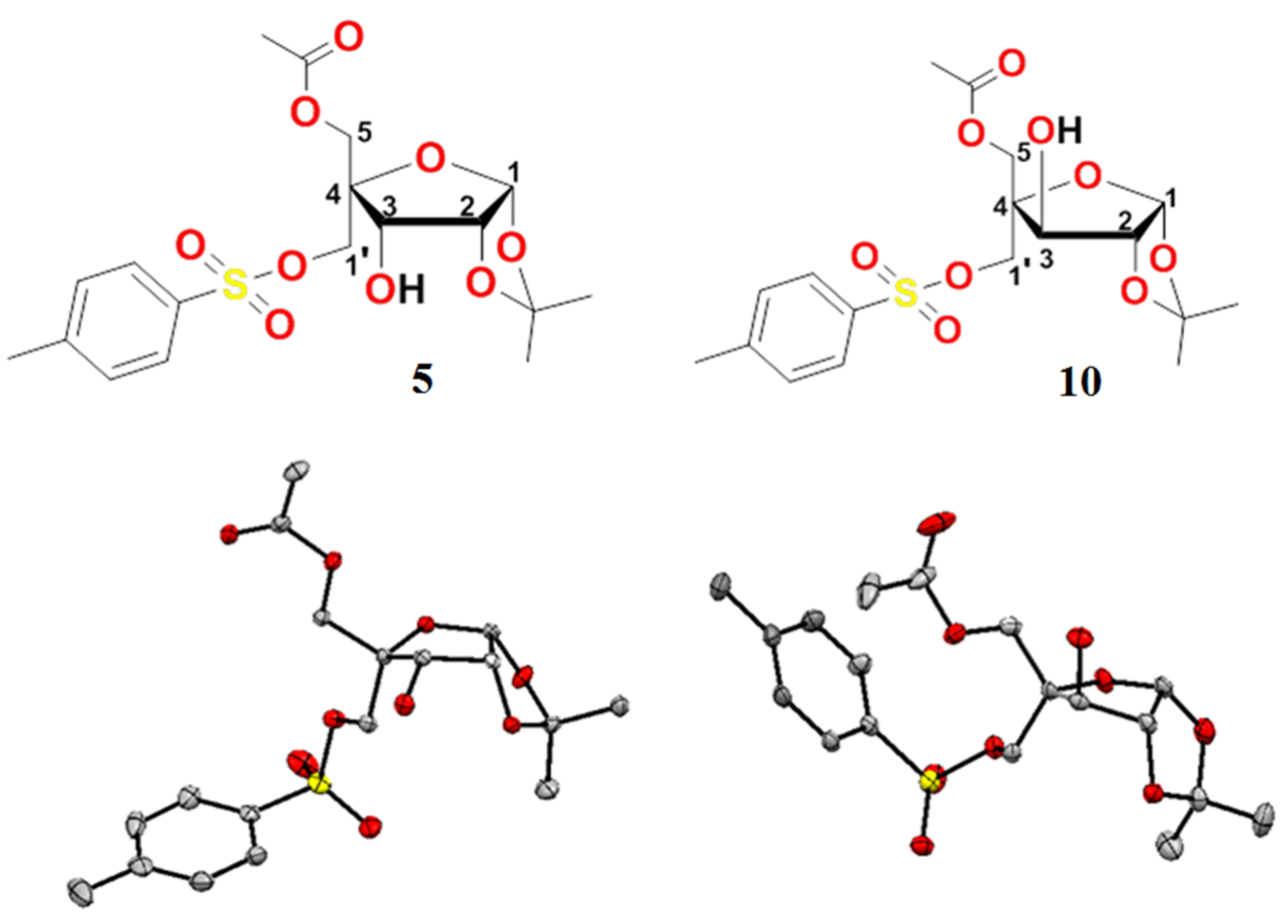
ORTEP diagram of tosylated sugar derivatives **5** and **10**.

**Scheme 3:**
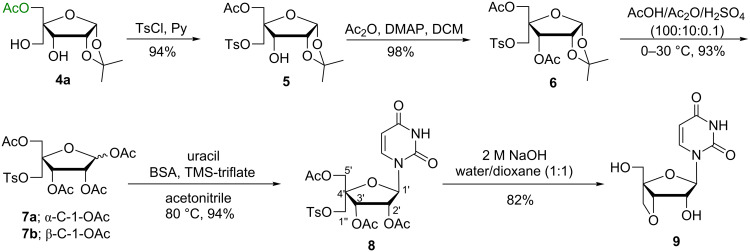
Convergent synthesis of 3′-*O*,4′-*C*-methyleneuridine.

**Scheme 4:**
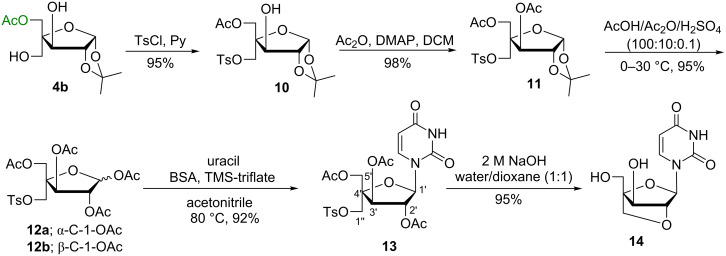
Convergent synthesis of 2′-*O*,4′-*C*-methylene-xylouridine.

The exclusive selectivity of the Novozyme^®^-435 for the acetylation of the C-5 hydroxy group of ribo*-*/xylotrihydroxyfuranoses **3a** and **3b** have been confirmed by X-ray diffraction studies on the single crystal of their corresponding 4-*C*-*p*-toluenesulfonyloxymethyl derivatives, i.e., 5-*O*-acetyl-1,2-*O*-isopropylidene-4-*C*-*p*-toluenesulfonyloxymethyl-α-D-ribofuranose (**5**) and 5-*O*-acetyl-1,2-*O*-isopropylidene-4-*C*-*p*-toluenesulphonyloxymethyl-α-D-xylofuranose (**10**, Figure 2). The tosyl derivatives **5** and **10** of dihydroxyfuranosides **4a** and **4b** were obtained by their tosylation with TsCl-pyridine in 94 and 95% yields, respectively (Scheme 3 and Scheme 4).

Two tosylated sugar derivatives **5** and **10** have been successfully used for the convergent synthesis of 3′-*O*,4′-*C*-methyleneuridine (**9**) and 2′-*O*,4′-*C*-methylene-xylouridine (**14**) to illustrate the usefulness of the trihydroxyribo*-*/xylofuranose sugar derivatives separated by an enzymatic acetylation methodology (Scheme 3 and Scheme 4). The acetylation of the lone hydroxy group in monotosylated sugar derivative **5** was carried out with acetic anhydride and 4-dimethylaminopyridine (DMAP) in dichloromethane (DCM) to give 3,5-di-*O*-acetyl-1,2-*O*-isopropylidene-4-*C*-*p*-toluenesulphonyloxymethyl-α-D-ribofuranose (**6**) in 98% yield. The glycosyl donor **7a**,**b** was prepared from acetolysis of compound **6** with acetic acid/acetic anhydride/sulfuric acid (100:10:0.1) in 93% yield. The Vorbrüggen coupling [17] of **7a**,**b** with uracil in the presence of *N,O*-bis(trimethylsilyl)acetamide (BSA) and trimethylsilyltrifluoromethane sulfonate (TMS-triflate) in acetonitrile yielded the triacetylated nucleoside **8** in 94% yield. Subsequently, deacetylation of acetoxy groups in nucleoside **8** with 2 M NaOH solution in water/dioxane (1:1) also led to the concomitant cylization between suitably placed C-3′-OH and C-1′′-tosyl groups to afford 3′-*O*,4′-*C*-methyleneuridine (**9**) in 82% yield (Scheme 3) in similar manner as described in our earlier report [18].

**Figure 3:**
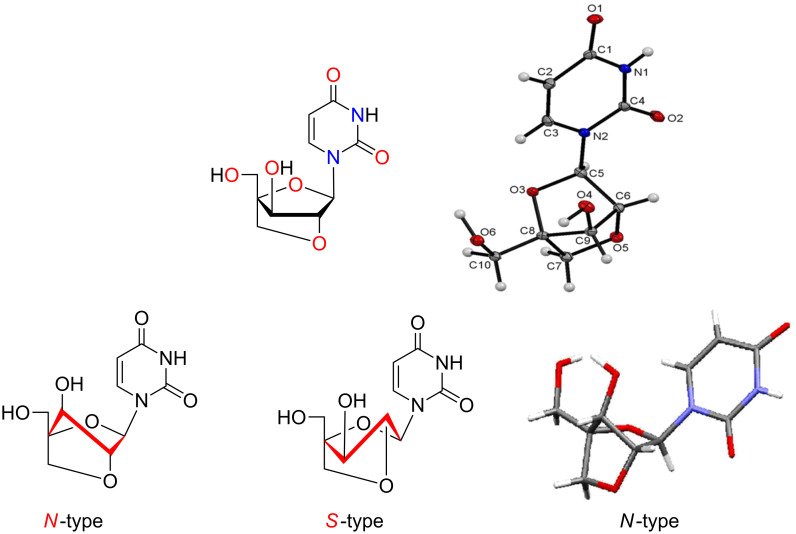
ORTEP diagram and preferential *N*-type sugar ring puckering of 2′-*O*,4′-*C*-methylene-xylouridine (**14**).

A similar sequence of reactions were performed for the synthesis of 2′-*O*,4′-*C*-methylene-xylouridine (**14**) from monotosylated sugar derivative **10** which, in turn, was obtained from enzymatic product **4b** in 95% yield. Thus, the acetylation of the lone hydroxy group of **10** using acetic anhydride and DMAP in dichloromethane afforded 3,5-di-*O*-acetyl-1,2-*O*-isopropylidene-4-*C*-*p*-toluenesulfonyloxymethyl-α-D-xylofuranose (**11**) in 98% yield. Acetolysis of compound **11** yielded the glycosyl donor **12a**,**b** in 95% yield, which on Vorbrüggen coupling with uracil under earlier used base-coupling conditions afforded the corresponding acetylated nucleoside **13** in 92% yield. Subsequent deacetylation of nucleosides **13** followed by concomitant cyclization with 2 M NaOH solution in water/dioxane (1:1) afforded 2′-*O*,4′-*C*-methylene-xylouridine (**14**) in 95% yield (Scheme 4) in a similar manner as described in our earlier report [18]. The structure of compound 2′-*O*,4′-*C*-methylene-xylouridine (**14**) was confirmed by X-ray diffraction studies on its single crystal which also confirms the possibility of restriction of ring puckering in bicyclic nucleoside and the sugar ring puckering is locked in *N*-type conformation (Figure 3).

The structures of all the synthesized compounds, i.e., **4a**, **4b**, **5**, **6**, **7a**,**b**, **8–11**, **12a**,**b**, **13** and **14** were unambiguously established on the basis of their spectral data (^1^H and ^13^C NMR spectra, IR spectra and HRMS) analysis. The structure of known compounds **9** [19] and **14** [20] were further confirmed on the basis of comparison of its physical and spectral data with those reported in the literature. The single crystal X-ray diffraction analysis has been performed on compounds **5**, **10** and **14** and their detailed crystallographic data have been deposited in the Cambridge Crystallographic Data Centre with CCDC No. 1533725, 1533768 and 1532373, respectively.

## Conclusion

A highly efficient and diastereoselective catalyzed Novozyme^®^-435-catalyzed acetylation methodology has been developed for the selective acetylation of one of the two diastereotopic primary hydroxymethyl groups present in the inseparable epimeric mixture of trihydroxyribo*/*xylofuranose. The biocatalytic selective acetylation of inseparable mixtures of ribo*/*xylofuranose derivatives has resulted in the easy separation of the 5-*O-*acetylated ribo*/*xylofuranose derivatives in quantitative yields. The sugar precursors separated by the biocatalytic methodology have been used for the convergent synthesis of the bicyclic nucleosides, 3′-*O*,4′-*C*-methyleneuridine and 2′-*O*,4′-*C*-methylene-xylouridine in 66% and 77% overall yields, respectively, from respective biaocatalytic monoacetylated product. The crystal structure of one of the synthesized bicyclic nucleosides, 2′-*O*,4′-*C*-methylene-xylouridine (**14**) revealed *N*-type puckering of the sugar ring of the compound. The developed biocatalytic methodology will have significant impact in the area of synthetic carbohydrate and nucleoside chemistry.

## Experimental

The *Candida antarctica* lipase-B (CAL-B or Novozyme^®^-435) immobilized on polyacrylate was purchased from Sigma-Aldrich Co. (USA). *Theremomyces lanuginosus* lipase (Lipozyme TL IM) immobilized on silica was obtained as a gift from Novozymes Inc., Copenhagen, Denmark. For all the lipase-mediated reactions, AR grade organic solvents were used, which were purchased from SD Fine-Chem Ltd., Mumbai, India. The IR spectra were recorded by using thin film for oils and by making KBr discs for solid samples. The ^1^H and ^13^C NMR spectra were recorded on a JEOL Alpha-400 spectrometer at 400 and 100.6 MHz, respectively, using TMS as internal standard. The chemical shift values are on δ scale and the coupling constants (*J*) are in Hz. The HRMS analysis was done on a Q-TOF mass spectrometer using ESI positive mode. The optical rotations were measured using light of 589 nm wavelength. Analytical TLCs were performed on precoated silica-gel 60 F_254_ plates; the spots were detected either under UV light or by charring with a solution of 4% H_2_SO_4_ in ethanol. Silica gel (100–200 mesh) was used for column chromatography. All solvents were distilled before use. The single crystal X-ray diffraction data was collected with graphite-monochromated Mo Kα radiation (λ = 0.71073 Å) at USIC, University of Delhi, Delhi.

**General procedure for biocatalytic acetylation of ribo*-* and xylotrihydroxyfuranosides 3a,b: synthesis of compounds 4a,b.** Similar as described in [18] to a solution of the mixture of compounds **3a**,**b** (2 g, 9.08 mmol) in 2-methyl-THF (40 mL), vinyl acetate (1.26 mL, 13.6 mmol) was added followed by the addition of Novozyme^®^-435 (0.2 g, 10% w/w of the compound **3a**,**b**). The reaction mixture was stirred at 35 °C in an incubator shaker for 1 h and the progress of the reaction was monitored periodically by TLC. On completion, the reaction was quenched by filtering off the enzyme, the solvent was removed under reduced pressure and the residue thus obtained was separated by column chromatography using methanol in chloroform as gradient solvent system to afford the two monoacetylated sugar derivatives **4a** and **4b**.

**5-*O*-Acetyl-4-*C*-hydroxymethyl-1,2-*O*-isopropylidene-α-D-ribofuranose** (**4a**). It was obtained as colourless oil (1.0 g, 42% yield). *R*_f_ = 0.4 (5.0% methanol in chloroform); [α]_D_^18^ +9.16 (*c* 0.1, MeOH); IR (thin film) ν_max_: 3471, 2946, 1739, 1383, 1244, 1166, 1046 875 cm^−1^; ^1^H NMR (CDCl_3_, 400 MHz) δ 5.86 (d, *J* = 4.6 Hz, 1H), 4.73–4.70 (m, 1H), 4.26–4.14 (m, 3H), 3.84 (s, 2H), 3.19 (d, *J* = 8.4 Hz, 1H), 2.61 (s, 1H), 2.10 (s, 3H), 1.62 (s, 3H), 1.39 (s, 3H); ^13^C NMR (CDCl_3_, 100.6 MHz) δ 170.73, 113.57, 104.68, 86.94, 79.41, 72.86, 65.73, 62.25, 26.45, 26.22, 20.85; HR-ESI-TOF-MS *m*/*z*: [M + Na]^+^ calcd. for [C_11_H_18_O_7_Na]^+^ 285.0945, found: 285.0947,

**5-*O*-Acetyl-4-*C*-hydroxymethyl-1**,**2-*O*-isopropylidene-α-D-xylofuranose** (**4b**). It was obtained as colourless oil (1.10 g, 46% yield). *R*_f_ = 0.3 (5.0% methanol in chloroform); [α]_D_^22^ −13.85 (*c* 0.1, MeOH); IR (thin film) ν_max_: 3446, 2943, 1739, 1377, 1248, 1164, 1048, 862 cm^−1^; ^1^H NMR (CDCl_3_, 400 MHz) δ 5.93 (d, *J* = 4.4 Hz, 1H), 4.64–4.63 (m, 1H), 4.31–4.19 (m, 3H), 3.77 (d, *J* = 11.2 Hz, 1H), 3.63 (d, *J* = 4.8 Hz, 1H), 2.82 (s, 1H), 2.15 (s, 1H), 2.11 (s, 3H), 1.53 (s, 3H), 1.30 (s, 3H); ^13^C NMR (CDCl_3_, 100.6 MHz) δ 170.76, 113.56, 104.64, 86.87, 79.44, 72.84, 65.70, 62.23, 26.43, 26.20, 20.84; HR-ESI-TOF-MS *m*/*z*: [M + H]^+^ calcd. for [C_11_H_19_O_7_]^+^ 263.1125, found 263.1130.

**General procedure for the tosylation of monoacetylated sugar derivatives 4a and 4b: synthesis of compounds 5 and 10.** Similar as described in [18] to a stirred solution of compound **4a** (2 g, 7.6 mmol) in pyridine (20 mL), *p*-toluenesulfonyl chloride (2.18 g, 11.4 mmol) was added at 0 °C. The progress of the reaction was monitored by TLC and on completion after 2 h, the reaction mixture was neutralized by 10% ice-cold hydrochloric acid solution (80 mL) and extracted with chloroform (3 × 100 mL). The combined organic extract was washed with saturated aqueous NaHCO_3_ (2 × 100 mL), water (2 × 100 mL) and dried over anhydrous Na_2_SO_4_. The solvent was removed under reduced pressure and the residue thus obtained was purified by silica gel column chromatography using ethyl acetate in petroleum ether as gradient solvent system to afford the tosylated compound **5**. The similar procedure has been followed for the synthesis of compound **10** using **4b** as starting material.

**5-*O*-Acetyl-1,2-*O*-isopropylidene-4-*C*-*p*-toluenesulfonyloxymethyl-α-D-ribofuranose** (**5**). It was obtained as white solid (2.98 g, 94% yield). *R*_f_ = 0.6 (5.0% methanol in chloroform); mp: 94 °C; [α]_D_^28^ +11.98 (*c* 0.1, MeOH); IR (thin film) ν_max_: 3483, 2989, 1745, 1458, 1362, 1239, 1190, 1095, 985, 839 cm^−1^; ^1^H NMR (CDCl_3_, 400 MHz) δ 7.78 (d, *J* = 8.4 Hz, 2H), 7.32 (d, *J* = 8.4 Hz, 2H), 5.77 (d, *J* = 3.8 Hz, 1H), 4.64 (dd, *J* = 5.7 and 4.2 Hz, 1H), 4.38–4.02 (m, 5H), 2.71 (d, *J* = 6.9 Hz, 1H), 2.41 (s, 3H), 1.99 (s, 3H), 1.42 (s, 3H), 1.31 (s, 3H); ^13^C NMR (CDCl_3_, 100.6 MHz) δ 170.34, 144.92, 132.43, 129.79, 128.10, 113.81, 104.54, 84.37, 79.31, 73.06, 68.19, 64.75, 26.20, 21.60, 20.69; HR-ESI-TOF-MS *m/z*: [M + K]^+^, calcd. for [C_18_H_24_O_9_SK]^+^ 455.0773, found: 455.0768.

**5-*O*-Acetyl-1,2-*O*-isopropylidene-4-*C*-*p*-toluenesulfonyloxymethyl-α-D-xylofuranose** (**10**). It was obtained as white solid (3.01 g, 95% yield). *R*_f_ = 0.6 (5.0% methanol in chloroform); mp: 82 °C; [α]_D_^24^ −27.64 (*c* 0.1, MeOH); IR (thin film) ν_max_: 3464, 2928, 1742, 1363, 1176, 1037, 838 cm^−1^; ^1^H NMR (CDCl_3_, 400 MHz) δ 7.80 (d, *J* = 8.4 Hz, 2H), 7.36 (d, *J* = 7.6 Hz, 2H), 5.91 (d, *J* = 4.0 Hz, 1H), 4.60 (d, *J* = 3.6 Hz, 1H), 4.30-4.12 (m, 5H), 2.45 (s, 3H), 2.10 (d, *J* = 5.2 Hz, 1H), 1.97 (s, 3H), 1.35 (s, 3H), 1.27 (s, 3H); ^13^C NMR (CDCl_3_, 100.6 MHz) δ 171.29, 145.11, 132.24, 129.88, 128.12, 112.59, 105.32, 86.95, 86.55, 75.88, 67.79, 62.48, 26.10, 25.66, 21.60, 20.68; HR-ESI-TOF-MS: *m*/*z*: [M + H]^+^, calcd. for [C_18_H_25_O_9_S]^+^ 417.1214, found: 417.1218.

**General procedure for acetylation of the lone hydroxy group in compounds 5 and 10: synthesis of compounds 6 and 11.** To a solution of compound **5** (3 g, 7.2 mmol) in dichloromethane (30 mL) were added DMAP (176 mg, 1.44 mmol) and Ac_2_O (1.02 mL, 10.8 mmol) and the reaction mixture was stirred at 25–30 °C for 3 h. On completion, the mixture was diluted with cold water (25 mL) and extracted with ethyl acetate (3 × 100 mL). The combined organic layer was washed with cold water (2 × 50 mL), dried over sodium sulfate and concentrated under reduced pressure. The residue thus obtained was purified by column chromatography using ethyl acetate in petroleum ether as gradient solvent system to afford compound **6** as colourless oil in 98% yield. The similar procedure has been followed for the synthesis of compound **11**, which was obtained as colourless oil using **4b** as starting material in 98% yield. The spectral data and other details of compound **6** and **11** are given in Supporting Information File 1.

**General procedure for the acetolysis of compounds 6 and 11: synthesis of tetraacetate compounds 7a,b and 12a,b.** Similar as described in [18] acetic anhydride (6.2 mL, 65.43 mmol) and concentrated sulfuric acid (0.03 mL, 0.65 mmol) was added to a stirred solution of compound **6** (3 g, 6.5 mmol) in acetic acid (37.4 mL, 654.33 mmol) at 0 ^o^C and mixture was stirred for 6 h at 30 °C. On completion, the reaction was quenched by addition of water (200 mL) and the product was extracted with chloroform (3 × 100 mL). The combined organic layer was washed with sodium bicarbonate solution (2 × 100 mL), with cold water (2 × 100 mL) and then dried over sodium sulfate. The solvent was removed under reduced pressure and the residue thus obtained was purified on silica gel column chromatography using ethyl acetate in petroleum ether as gradient solvent system to afford an anomeric mixture **7a**,**b** as colourless viscous oil in 93% yield. A similar procedure has been followed for the synthesis of compound **12a**,**b** as colourless viscous oil using **11** as starting material in 95% yield. Detailed spectral data and other details of compounds **7a**,**b** and **12a**,**b** have been given in Supporting Information File 1.

**General procedure for the Vorbrüggen coupling of tetraacetylated sugar derivatives 7a,b and 12a,b: synthesis of acetylated nucleosides 8 and 13.** Similar as described in [18] to a stirred solution of compound **7a**,**b** (3.0 g, 5.97 mmol) and nucleobase uracil (1.0 g, 8.9 mmol) in anhydrous acetonitrile (25 mL), *N*,*O*-bis(trimethylsilyl)acetamide (5.9 mL, 23.88 mmol) was added dropwise. The reaction mixture was stirred at reflux for 1 h, and then cooled to 0 °C. Trimethylsilyltrifluoromethane sulfonate (1.8 mL, 10.14 mmol) was added dropwise into the cooled reaction mixture under stirring and the reaction mixture was refluxed for 4–6 h. The reaction was quenched with a cold saturated aqueous solution of sodium hydrogen carbonate (200 mL) and the reaction mixture was extracted with chloroform (3 × 100 mL). The combined organic phase was washed with saturated aqueous solutions of NaHCO_3_ (2 × 100 mL), brine (2 × 100 mL) and cold water (2 × 100 mL). The washed organic phase was then dried over anhydrous Na_2_SO_4_. The solvent was removed under reduced pressure and the residue thus obtained was purified by silica gel column chromatography using methanol in chloroform as gradient solvent system to afford nucleoside **8** as white solid in 94% yield. The similar procedure has been followed for the synthesis of compound **13**, which was afforded as white solid using **12a**,**b** as starting material in 92% yield. Detailed spectral data and other details of compounds **8** and **13** have been given in Supporting Information File 1.

**General procedure for the synthesis of bicyclic nucleosides 9 and 14.** Similar as described in [18] to a stirred solution of triacetylated nucleosides **8** (1 g, 1.8 mmol) in dioxane/water (1:1, 8 mL) was added 2 M NaOH solution (8 mL) and the reaction mixture was stirred at 30 °C for 2–10 h. On completion, the reaction mixture was neutralized with acetic acid and the solvent was removed under reduced pressure. The residue thus obtained was purified by silica gel column chromatography using methanol in chloroform as gradient solvent system to afford **9**. The similar procedure has been followed for the synthesis of compound **14** using triacetylated nucleoside **13** as starting material.

**3′-*O*,4′-*C*-Methyleneuridine** (**9**). It was obtained as white solid (0.38 g, 82% yield). *R*_f_ = 0.5 (10% methanol in chloroform); mp: 215–218 °C; [α]_D_^30^ –17.50 (*c* 0.1, MeOH); IR (thin film) ν_max_: 3371, 2832, 1685, 1461, 1262, 1025, 816 cm^−1^; ^1^H NMR (DMSO-*d*_6_, 400 MHz) δ 11.44 (s, 1H), 7.95 (d, *J* = 8.4 Hz, 1H), 6.14 (d, *J* = 3.1 Hz, 1H), 5.98 (s, 1H), 5.73 (d, *J* = 8.4 Hz, 1H), 5.37 (s, 1H), 4.90 (s, 1H), 4.62 (d, *J* = 7.6 Hz, 1H), 4.50 (d, *J* = 2.3 Hz, 1H), 4.13 (d, *J* = 6.9 Hz, 1H) 3.53 (s, 2H); ^13^C NMR (DMSO-*d*_6_, 100.6 MHz) δ 163.72, 151.26, 141.64, 102.90, 94.81, 91.28, 90.21, 79.22, 75.88, 61.26; HR-ESI-TOF-MS: *m*/*z*: [M + H]^+^, calcd. for [C_10_H_13_N_2_O_6_]^+^ 257.0768, found: 257.0760.

**2′-*O*,4′-*C*-Methylene-xylouridine** (**14**). It was obtained as white solid (0.43 g, 95% yield). *R*_f_ = 0.5 (10% methanol in chloroform); mp: 117–120 °C; [α]_D_^20^ +36.86 (*c* 0.1, MeOH); IR (thin film) ν_max_: 3370, 2946, 1680, 1460, 1271, 1022, 755 cm^−1^; ^1^H NMR (DMSO-*d*_6_, 400 MHz) δ 11.27 (s, 1H), 7.67 (d, *J* = 8.2 Hz, 1H), 5.69 (d, *J* = 1.8 Hz, 1H), 5.51–5.48 (m, 2H), 5.01 (t, *J* = 5.3 Hz, 1H), 4.21 (s, 1H), 4.06 (s, 1H), 3.96 (d, *J* = 8.2 Hz, 1H), 3.83–3.76 (m, 2H), 3.73 (d, *J* = 8.2 Hz, 1H); ^13^C NMR (DMSO-*d*_6_, 100.6 MHz) δ 163.56, 150.26, 141.43, 98.85, 89.72, 88.33, 77.52, 72.86, 72.00 56.86; HR-ESI-TOF-MS: *m*/*z*: [M + H]^+^, calcd. for [C_10_H_13_N_2_O_6_]^+^ 257.0768, found: 257.0769.

**X-ray diffraction studies on tosylated sugar derivatives 5 and 10 and bicyclic nucleoside 2′-*O*,4′-*C*-methylene-xylouridine (14).** Single crystal suitable for X-ray diffraction studies were grown by dissolving the tosylated sugar derivative **5** in toluene and the other tosylated sugar derivative **10** and 2′-*O*,4′-*C*-methylene-xylouridine (**14**) in methanol/chloroform and allowing slow evaporation of the solutions at room temperature. The X-ray diffraction data was collected with graphite monochromated Mo Kα radiation (λ = 0.71073 Å) at a temperature of 293 K. The structures were solved by direct methods using SHELXS-97 and refined by full-matrix least-squqres method on F2 (SHELXL-97) [20]. All calculations were carried out using the WinGX package of the crystallographic programs [21]. For the molecular graphics, the programs DIAMOND-2 [22] and Mercury [23–24] were used. Molecular structures have been drawn using ORTEP as software as given in Figure 2 and Figure 3. The selected bond lengths, bond angles, etc. are given in Table 1.

**Table 1:** Single crystal X-ray diffraction data of tosylated sugar derivatives **5**, **10** and 2′-*O*,4′-*C*-methylene-xylouridine (**14**).

	compound **5**	compound **10**	compound **14**

empirical formula	C_18_H_24_O_9_S	C_18_H_24_O_9_S	C_10_H_12_N_2_O_6_
formula weight	416.43	416.43	256.22
crystal system	monoclinic	monoclinic	monoclinic
space group	*P*21	*P*21	*P*21
unit cell dimensions	*a* = 8.3342(4) Å α = 90°	*a* = 11.354 Å α = 90°	*a* = 6.1313(3) Å α = 90°
	*b* = 11.6972(5) Å β = 95.660(4)°	*b* = 9.964 Å β = 98.66°	*b* = 7.3638(3) Å β = 93.213(4)°
	*c* = 10.4684(4) Å γ = 90°	*c* = 18.512 Å γ = 90°	*c* = 11.7021(6) Å γ = 90°
volume	1015.56(8) Å^3^	2070.4 Å^3^	527.52(4) Å^3^
Z	2	2	2
density	1.362 mg/m^3^	1.362 mg/m^3^	1.613 mg/m^3^
absorption coefficient	0.206 mm^−1^	0.205 mm^−1^	0.135 mm^−1^
F(000)	440	896	268
index ranges	−9<=h<=6, −13<=k<=13, −12<=l<=12	−13<=h<=13, −11<=k<=11, −19<=l<=22	−6<=h<=7, −8<=k<=8, −10<=l<=13
R(int)	0.0130	0.0256	0.0140
GOF on F2	1.030	1.022	1.030
final R indices	R1 = 0.0311	R1 = 0.0500	R1 = 0.0288
I>2sigma(I)	wR2 = 0.0720	wR2 = 0.1070	wR2 = 0.0691
R indices	R1 = 0.0348	R1 = 0.0647	R1 = 0.0298
all data	wR2 = 0.0738	wR2 = 0.1150	wR2 = 0.0701
CCDC	1533725	1533768	1532373

## Supporting Information

File 1 Additional analytical data and NMR spectra.
