# Supplementary material for: Enzymatic separation of epimeric 4-C-hydroxymethylated furanosugars: Synthesis of bicyclic nucleosides
Source: Beilstein J Org Chem. 2017 Oct 5;13:2078–86. doi: 10.3762/bjoc.13.205 (PMC5647706; doi:10.3762/bjoc.13.205)
Supplement: File 1 — Additional analytical data and NMR spectra. [file Beilstein_J_Org_Chem-13-2078-s001.pdf]

Supporting Information

for

Enzymatic separation of epimeric 4-*C*-hydroxymethylated furanosugars: Synthesis of bicyclic nucleosides

Neha Rana, Manish Kumar, Vinod Khatri, Jyotirmoy Maity and Ashok K. Prasad*

Address: Bioorganic Laboratory, Department of Chemistry, University of Delhi, Delhi-110 007, India; Phone: 00-91-11-27662486

Email: Ashok K. Prasad - ashokenzyme@gmail.com
*Corresponding author

Additional analytical data and NMR spectra

Table of Contents

Characterization data : S3–S6

Fig. S1: ^{1}H NMR spectrum of compound **4a...S7**

Fig. S2: ^{13}C NMR spectrum of compound **4a..S7**

Fig. S3: ^{1}H NMR spectrum of compound **4b...S8**

Fig. S4: ^{13}C NMR spectrum of compound **4b..S8**

Fig. S5: ^{1}H NMR spectrum of compound **5..S9**

Fig. S6: ^{13}C NMR spectrum of compound **5...S9**

Fig. S7: ^{1}H NMR spectrum of compound **10...S10**

Fig. S8: ^{13}C NMR spectrum of compound **10..S10**

Fig. S9: ^{1}H NMR spectrum of compound **6..S11**

Fig. S10: ^{13}C NMR spectrum of compound **6...S11**

Fig. S11: ^{1}H NMR spectrum of compound **11..S12**

Fig. S12: ^{13}C NMR spectrum of compound **11...S12**

Fig. S13: ^{1}H NMR spectrum of compound **7a,b**..S13

Fig. S14: ^{13}C NMR spectrum of compound **7a,b**...S13

Fig. S15: ^{1}H NMR spectrum of compound **12a,b**...S114

Fig. S16: ^{13}C NMR spectrum of compound **12a,b**...S14

Fig. S17: ^{1}H NMR spectrum of compound **8**...S15

Fig. S18: ^{13}C NMR spectrum of compound **8**..S15

Fig. S19: ^{1}H NMR spectrum of compound **13**..S16

Fig. S20: ^{13}C NMR spectrum of compound **13**...S16

Fig. S21: ^{1}H NMR spectrum of compound **9** S17

Fig. S22: ^{13}C NMR spectrum of compound **9** S17

Fig. S23: ^{1}H NMR spectrum of compound **14**..S18

Fig. S24: ^{13}C NMR spectrum of compound **14**...S18

3,5-Di-*O*-acetyl-1,2-*O*-isopropylidene-4-*C*-*p*-toluenesulfonyloxymethyl-α-D-ribofuranose (6).

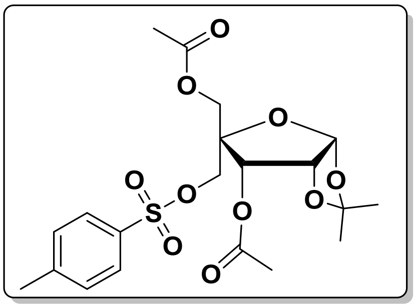

It was obtained as colourless oil (3.24 g, 98 % yield). R_f = 0.6 (3.0 % methanol in chloroform); $[\alpha]_D^{26}$ = +5.04 (*c* 0.1, MeOH); IR (thin film) ν_{max}: 2988, 1748, 1647, 1461, 1368, 1223, 1179, 1028, 989 and 836 cm^{-1}; ^1H NMR ($CDCl_3$, 400 MHz): δ 7.78 (dd, *J* = 8.0, 1.9 Hz, 2H), 7.33 (d, *J* = 6.9 Hz, 2H), 5.77-5.75 (m, 1H), 5.07-5.06 (m, 1H), 4.85-4.72 (m, 1H), 4.43 (dd, *J* = 10.3 and 1.9 Hz, 1H), 4.26 (dd, *J* = 10.3 and 1.9 Hz, 1H), 4.12 (dd, *J* = 12.2 and 2.3 Hz, 1H), 4.04 (dd, *J* = 12.2 and 2.3 Hz, 1H), 2.42 (s, 3H), 2.12 (s, 3H), 1.97 (s, 3H), 1.39 (s, 3H), 1.27 (s, 3H); ^{13}C NMR ($CDCl_3$, 100.6 MHz): δ 168.57, 168.33, 143.59, 131.10, 128.43, 126.59, 112.61, 103.00, 81.26, 77.00, 71.50, 66.32, 63.08, 24.84, 24.60, 20.18, 19.19, 19.07; HR-ESI-TOF-MS: *m/z* 476.1594 ($[M+NH_4]^+$), calcd. for $[C_{20}H_{26}O_{10}S+NH_4]^+$ 476.1585.

3,5-Di-*O*-acetyl-1,2-*O*-isopropylidene-4-*C*-*p*-toluenesulfonyloxymethyl-α-D-xylofuranose (11).

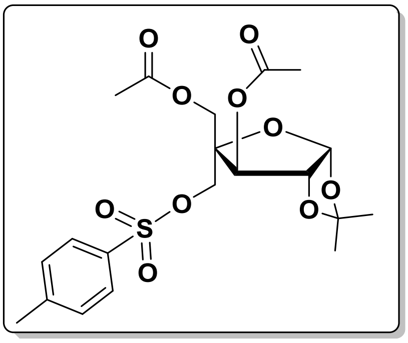

It was obtained as colorless oil (3.24 g, 98 % yield). $R_f = 0.5$ (3.0 % methanol in chloroform); $[\alpha]_D^{31} = -22.08$ (*c* 0.1, MeOH); IR (thin film) ν_{max}: 2988, 1748, 1598, 1459, 1368, 1219, 1180, 1056, 986, 835 cm^{-1}; ^1H NMR ($CDCl_3$, 400 MHz): δ 7.81 (d, *J* = 8.4 Hz, 2H), 7.36 (d, *J* = 8.4 Hz, 2H), 5.92 (d, *J* = 3.8 Hz, 1H), 5.25 (s, 1H), 4.52 (d, *J* = 3.8 Hz, 1H), 4.31-4.05 (m, 4H), 2.45 (s, 3H), 2.09 (s, 3H), 1.95 (s, 3H), 1.39 (s, 3H), 1.26 (s, 3H); ^{13}C NMR ($CDCl_3$, 100.6 MHz): δ 170.02, 169.10, 145.10, 132.24, 129.87, 128.13, 113.09, 105.43, 85.44, 85.14, 67.14, 61.77, 26.13, 25.72, 21.61, 20.58; HR-ESI-TOF-MS: *m/z* 459.1323 ($[M+H]^+$), calcd. for $[C_{20}H_{26}O_{10}S+H]^+$ 459.1319.

1,2,3,5-Tetra-*O*-acetyl-4-*C*-*p*-toluenesulfonyloxymethyl-α,β-D-ribofuranose (7a,b).

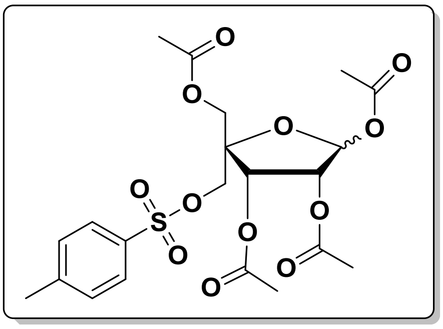

It was obtained as colourless viscous oil (3.04 g, 93 % yield). $R_f = 0.5$ (2 % methanol in choloroform). IR (thin film) ν_{max}: 2964, 1753, 1598, 1436, 1371, 1294, 1178, 1046, 840 cm^{-1}; ^1H NMR ($CDCl_3$, 400 MHz): δ 7.79, 7.37, 6.06 (s, 1H), 5.51, 5.35, 4.32-4.22, 4.10, 2.46, 2.20-2.06 1.94 (s, 3H); ^{13}C NMR ($CDCl_3$, 100.6 MHz): δ 169.80, 169.25, 169.09, 168.70, 145.19, 132.22, 129.94, 129.80, 128.03, 127.91, 91.19, 82.72, 71.36, 66.90, 64.55, 21.62, 20.92, 20.49, 20.43, 20.25; HR-ESI-TOF-MS: *m/z* 525.1047 ($[M+Na]^+$), calcd. for $[C_{21}H_{26}O_{12}S+Na]^+$ 525.1037.

1,2,3,5-Tetra-*O*-acetyl-4-*C*-*p*-toluenesulfonyloxymethyl-α,β-D-xylofuranose (12a,b).

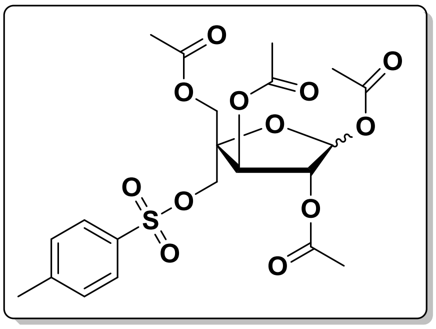

It was obtained as colourless viscous oil (3.12 g, 95 % yield). R_f = 0.4 (2.0 % methanol in choloroform). IR (thin film) ν_{max} 2963, 1753, 1676, 1598, 1495, 1437, 1370, 1294, 1221, 1178, 1053 838 cm^{-1}; ^1H NMR ($CDCl_3$, 400 MHz): δ 7.84-7.76, 7.37, 6.38-6.33 (m), 6.06, 5.52-5.44, 5.37 (m), 5.32, 5.16, 4.41 , 4.25-4.16, 4.10-3.92, 2.46, 2.13-1.96; ^{13}C NMR ($CDCl_3$, 100.6 MHz): δ 169.97, 169.93, 169.79, 169.24, 169.16, 169.06, 169.01, 145.26, 145.15, 132.34, 132.19, 129.89, 128.02, 96.64, 91.77, 85.74, 81.57, 80.56, 74.17, 69.79, 68.08, 61.93, 61.95, 21.65, 20.99, 20.91, 20.71, 20.51 and 20.40; HR-ESI-TOF-MS: *m/z* 525.1054 ($[M+Na]^+$), calcd. for $[C_{21}H_{26}O_{12}S+Na]^+$ 525.1037.

2′,3′,5′-Tri-*O*-acetyl-4′-*C*-*p*-toluenesulfonyloxymethyluridine (8).

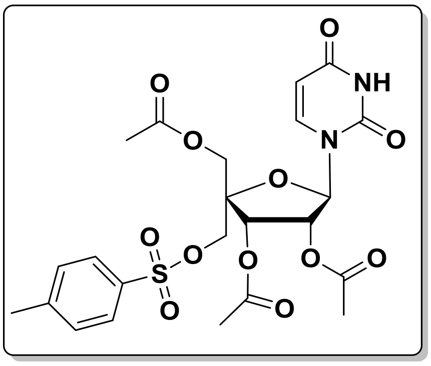

It was obtained as white solid (3.1 g, 94 % yield). R_f = 0.5 (5.0 % methanol in chloroform); M. Pt.: 114 ^oC; $[\alpha]_D^{22}$ = -15.72 (*c* 0.1, MeOH); IR (thin film) ν_{max}: 2923, 2362, 1752, 1699, 1458, 1375, 1299, 1229, 1053, 816 cm^{-1}; ^1H NMR ($CDCl_3$, 400 MHz): δ 9.22 (s, 1H), 7.76 (d, *J* = 8.4 Hz, 2H), 7.35 (d, *J* = 7.6 Hz, 2H), 7.24 (s, 1H), 5.85 (d, *J* = 5.3 Hz, 1H), 5.74 (d, *J* = 8.4 Hz, 1H), 5.54 (d, *J* = 6.1 Hz, 1H), 5.49-5.41 (m, 1H), 4.33 (d, *J* = 12.2 Hz, 1H), 4.23 (d,

J = 9.9 Hz, 1H), 4.12 (m, 2H), 2.43 (s, 3H), 2.18-2.01 (m, 9H); ^{13}C NMR ($CDCl_3$, 100.6 MHz): δ 169.73, 169.65, 168.96, 162.63, 149.97, 145.46, 140.07, 132.23, 129.99, 127.93, 103.40, 88.52, 83.31, 72.48, 70.67, 66.75, 64.33, 21.66, 20.64, 20.37, 20.27; HR-ESI-TOF-MS: m/z 577.1085 ([M+Na]$^+$), calcd. for $[C_{23}H_{26}N_2O_{12}S+Na]^+$ 577.1099.

2′,3′,5′-Tri-*O*-acetyl-4′-*C*-*p*-toluenesulfonyloxymethylxylouridine (**13**).

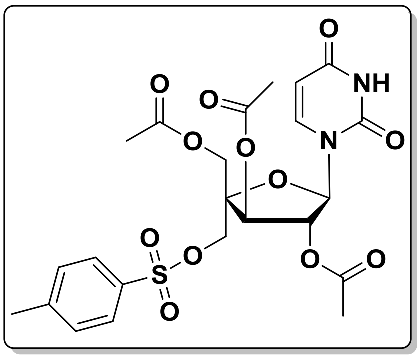

It was obtained as white solid **(**3.04 g, 92 % yield). R_f = 0.5 (5.0 % methanol in chloroform); M. Pt.: 127 ^{o}C; $[\alpha]_D^{31}$ = +18.32 (*c* 0.1, MeOH); IR (thin film) ν_{max}: 2943, 2374, 1720, 1621, 1473, 1320, 1300, 1201, 1078, and 854 cm^{-1}; ^{1}H NMR ($CDCl_3$, 400 MHz): δ 9.07 (s, 1H), 7.79 (d, J = 8.4 Hz, 2H), 7.45 (d, J = 7.6 Hz, 1H), 7.35 (d, J = 8.4 Hz, 2H), 6.00 (d, J = 6.1 Hz, 1H), 5.78 (d, J = 7.6 Hz, 1H), 5.45 (d, J = 5.3 Hz, 1H), 5.34 (t, J = 5.7 Hz, 1H), 4.45 (d, J = 12.2 Hz, 1H), 4.24-4.10 (m, 2H), 3.97 (d, J = 12.2 Hz, 1H), 2.43 (s, 3H), 2.10-2.01 (m, 9H); ^{13}C NMR ($CDCl_3$, 100.6 MHz): δ 169.76, 169.56, 169.29, 149.97, 145.50, 138.75, 132.03, 130.14, 130.01, 128.05, 127.91, 103.55, 85.87, 83.19, 74.87, 68.43, 61.73, 21.67, 20.72, 20.48, 20.43; HR-ESI-TOF-MS: m/z 572.1544 ([M+NH_4]$^+$), calcd. for $[C_{23}H_{26}N_2O_{12}S+ NH_4]^+$ 572.1545.

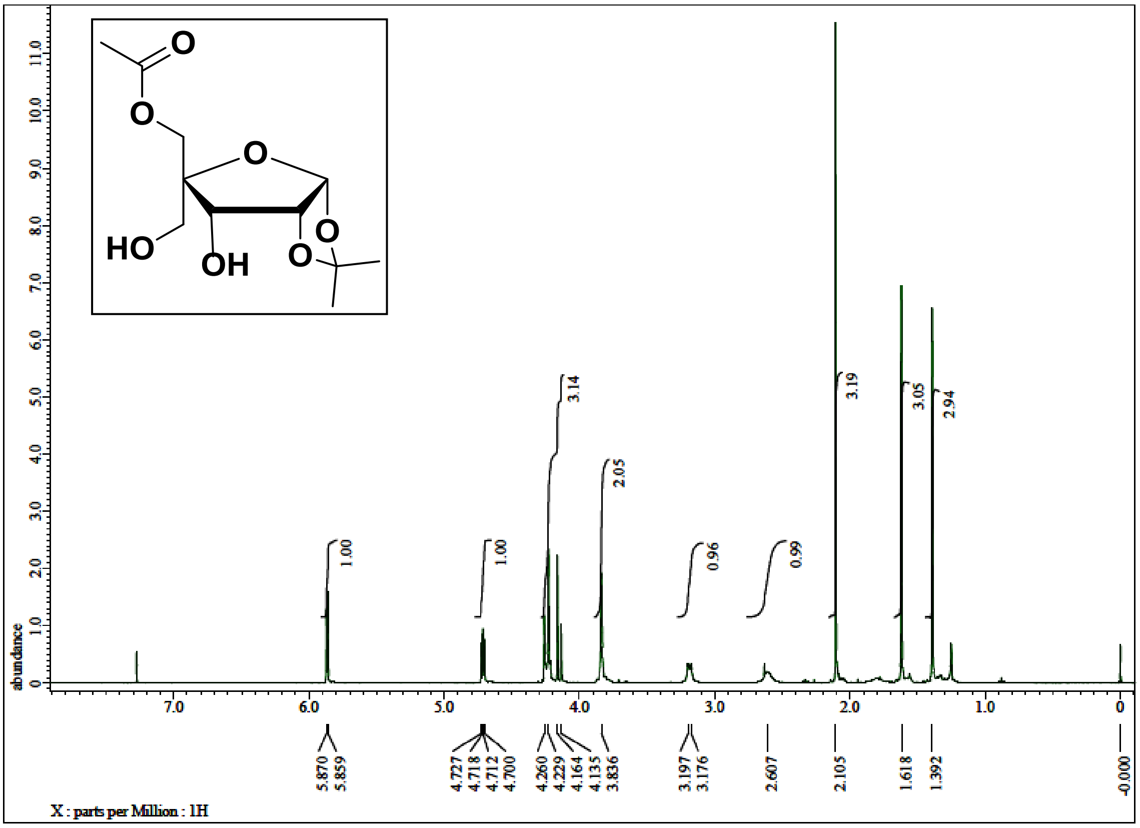

Figure S1: ^{1}H NMR spectrum of compound **4a** (400 MHz, $CDCl_3$)

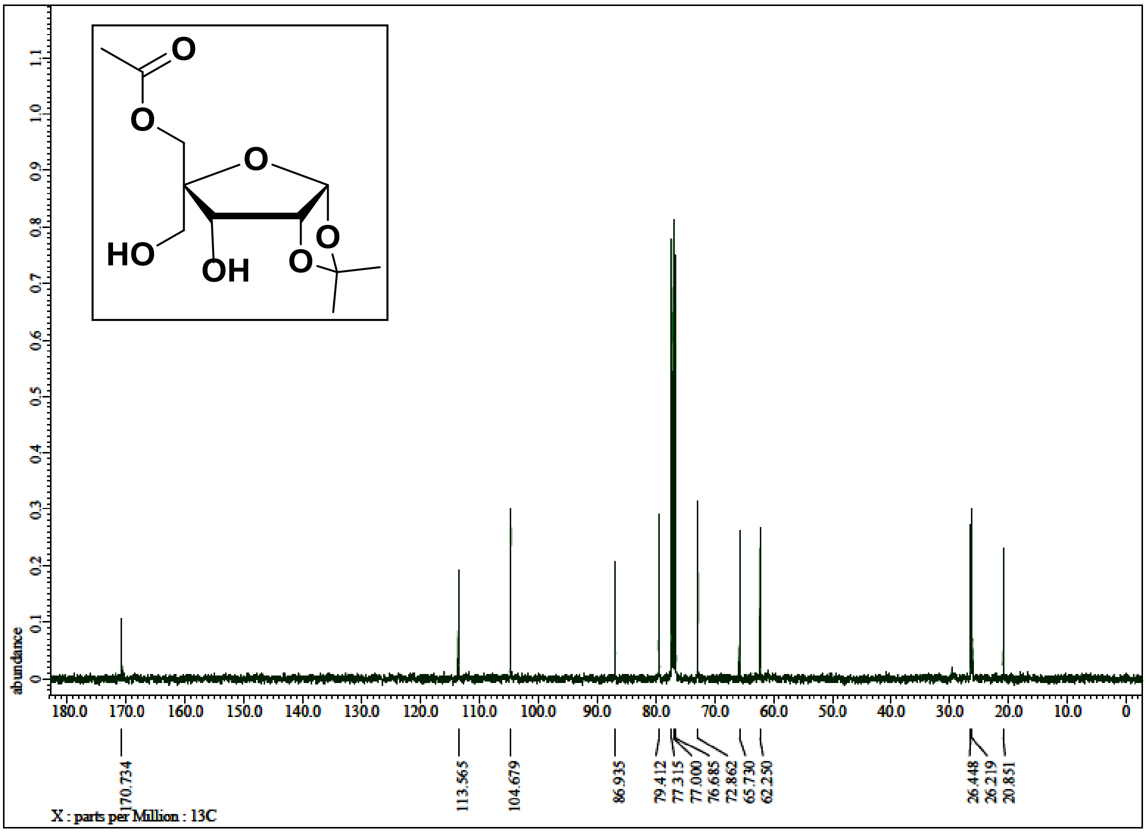

Figure S2: ^{13}C NMR spectrum of compound **4a** (100.6 MHz, $CDCl_3$)

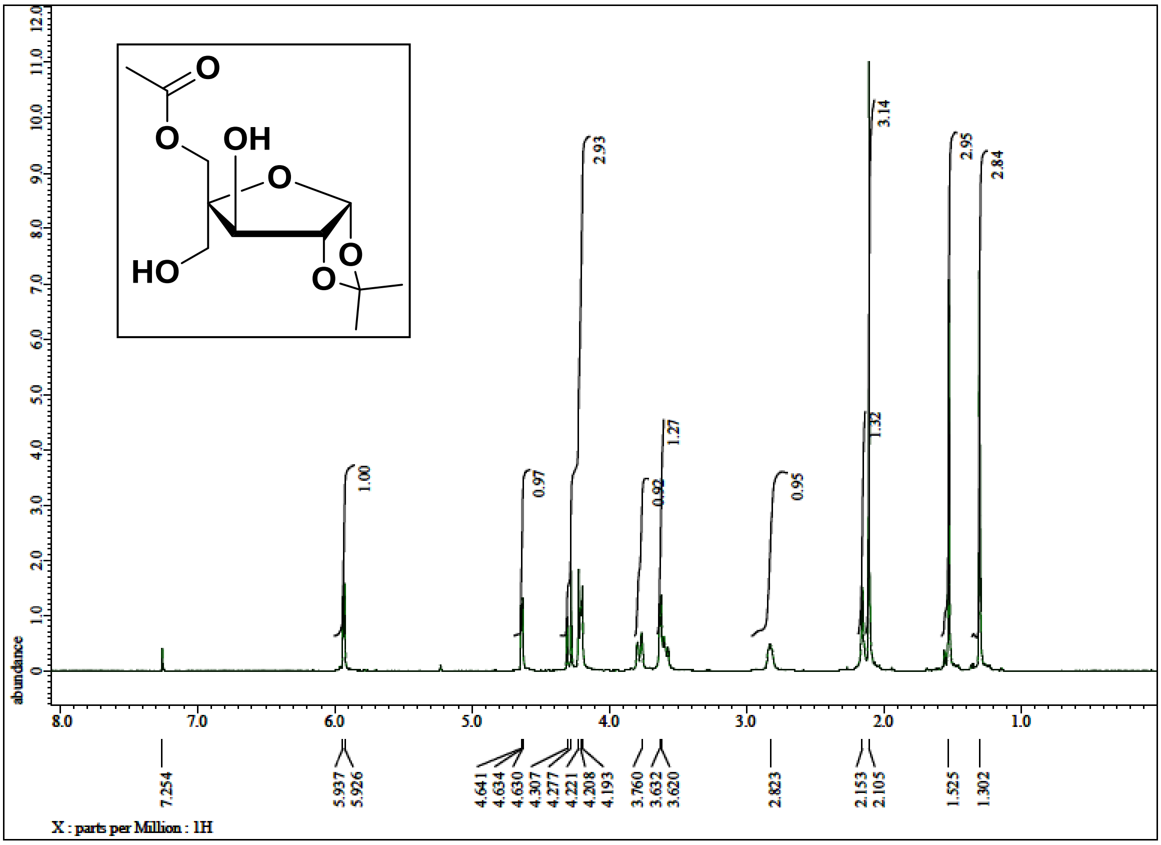

Figure S3: ^{1}H NMR spectrum of compound **4b** (400 MHz, $CDCl_3$)

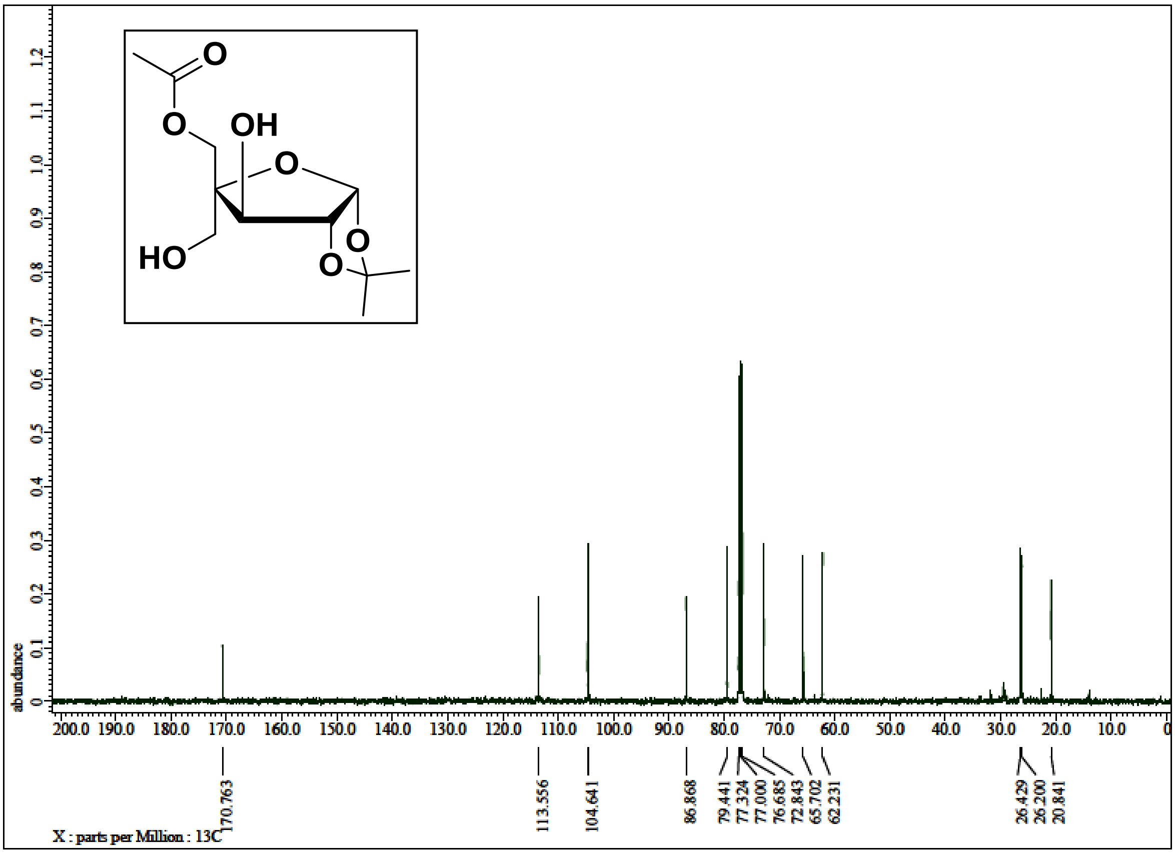

Figure S4: ^{13}C NMR spectrum of compound **4b** (100.6 MHz, $CDCl_3$)

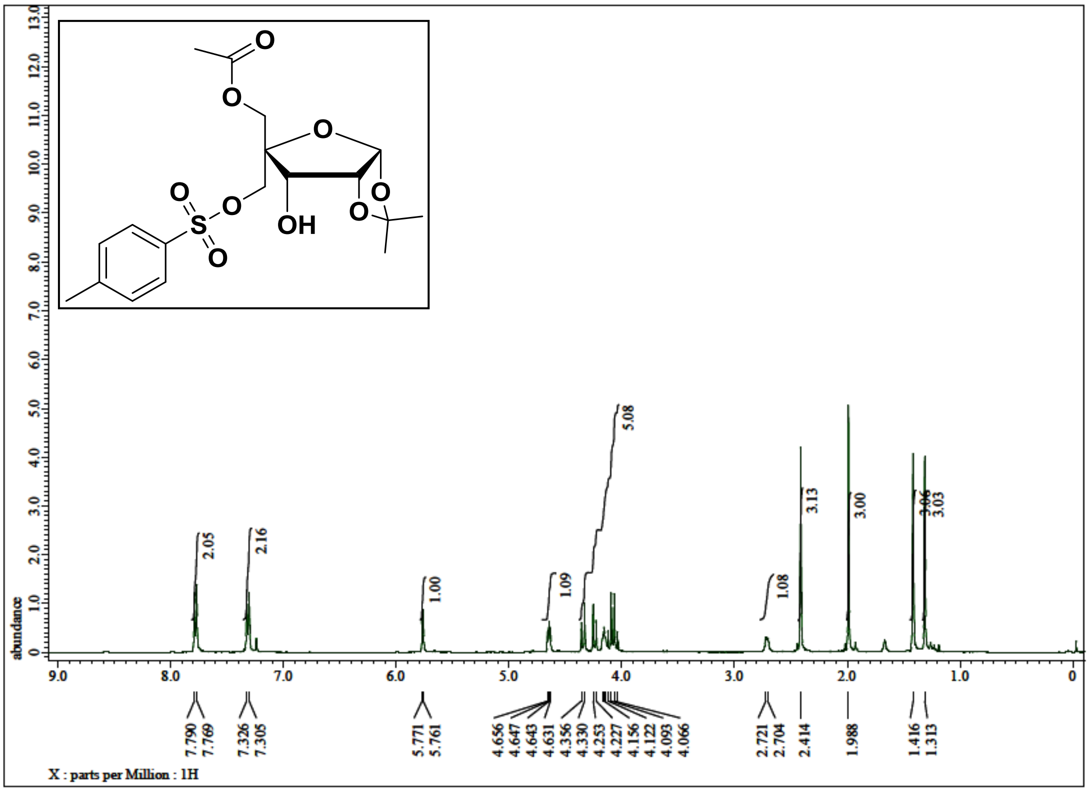

Figure S5: ^{1}H NMR spectrum of compound **5** (400 MHz, $CDCl_3$)

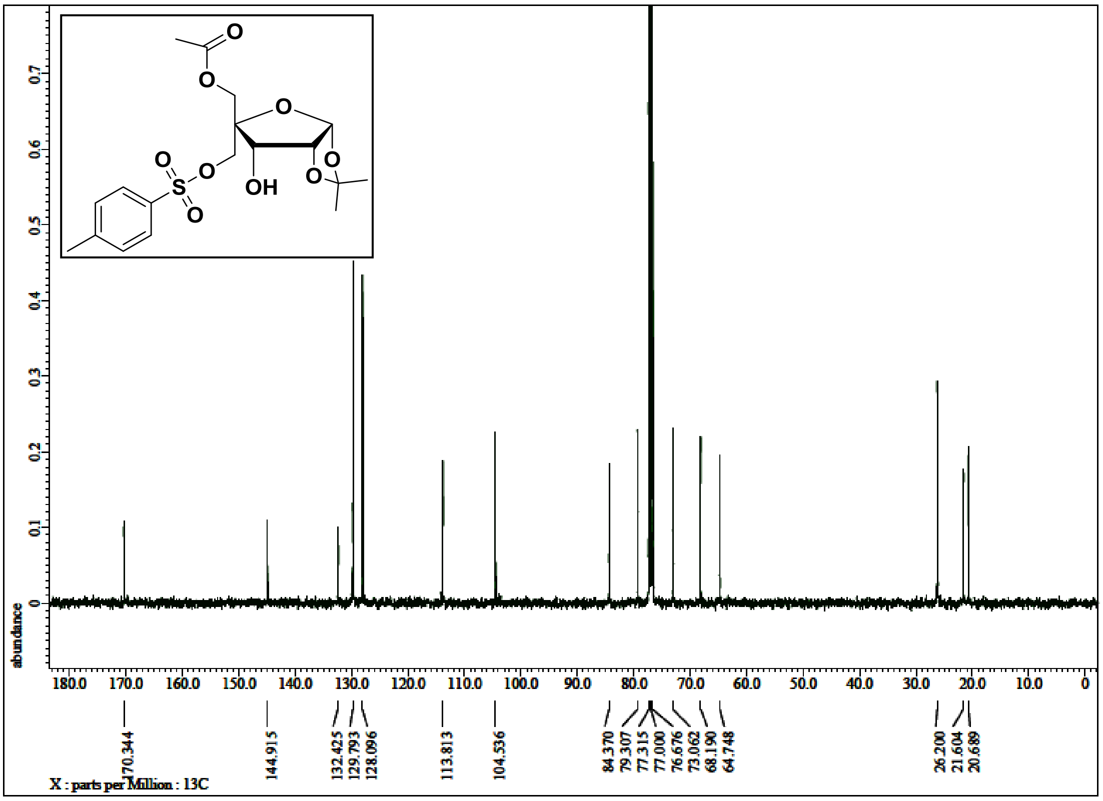

Figure S6: ^{13}C NMR spectrum of compound **5** (100.6 MHz, $CDCl_3$)

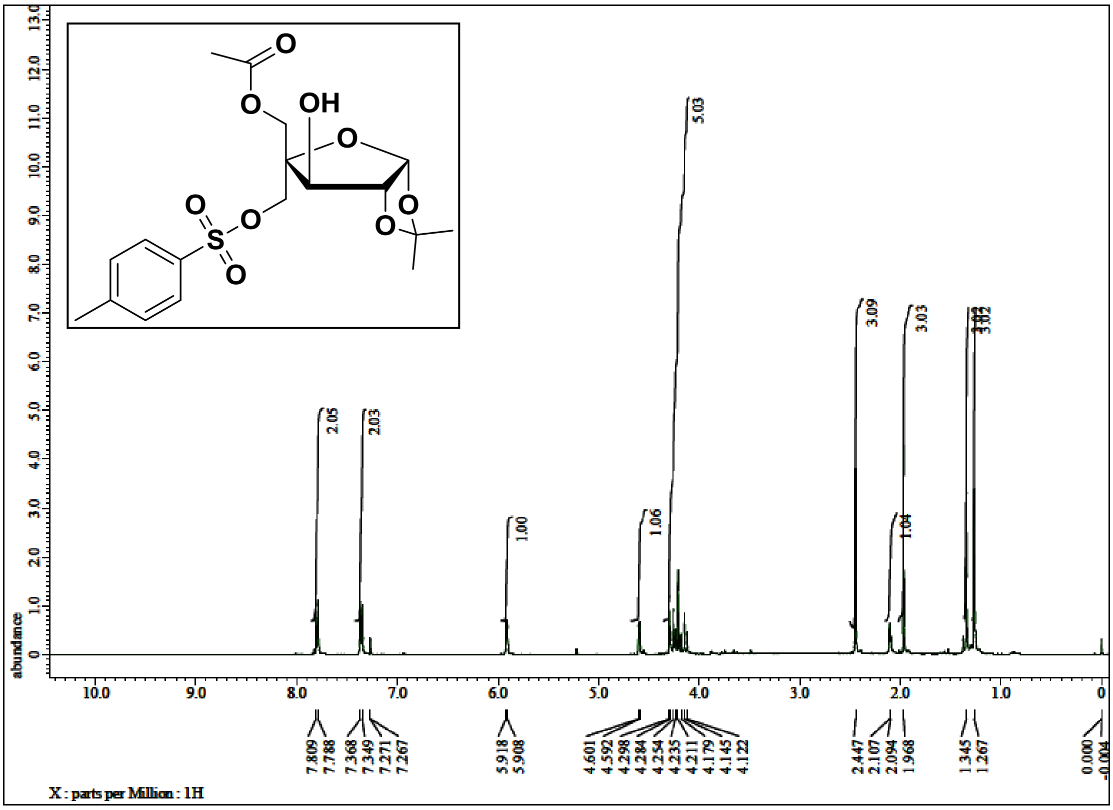

Figure S7: ^{1}H NMR spectrum of compound **10** (400 MHz, $CDCl_3$)

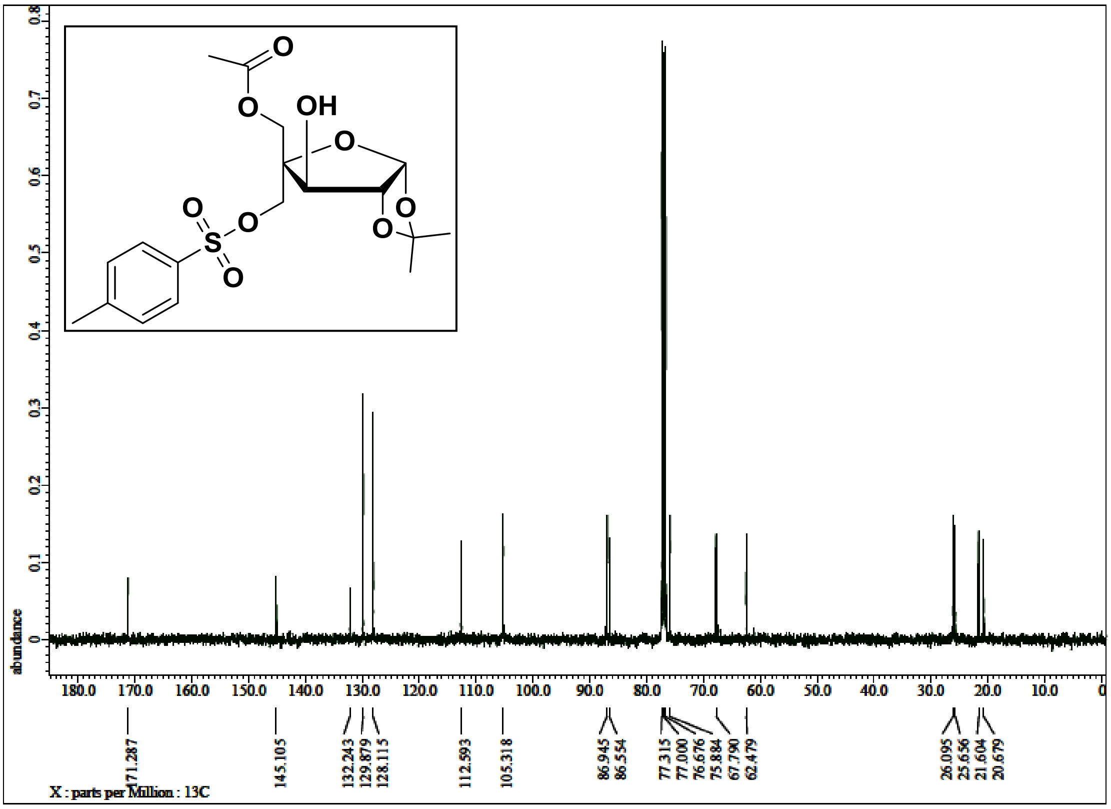

Figure S8: ^{13}C NMR spectrum of compound **10** (100.6 MHz, $CDCl_3$)

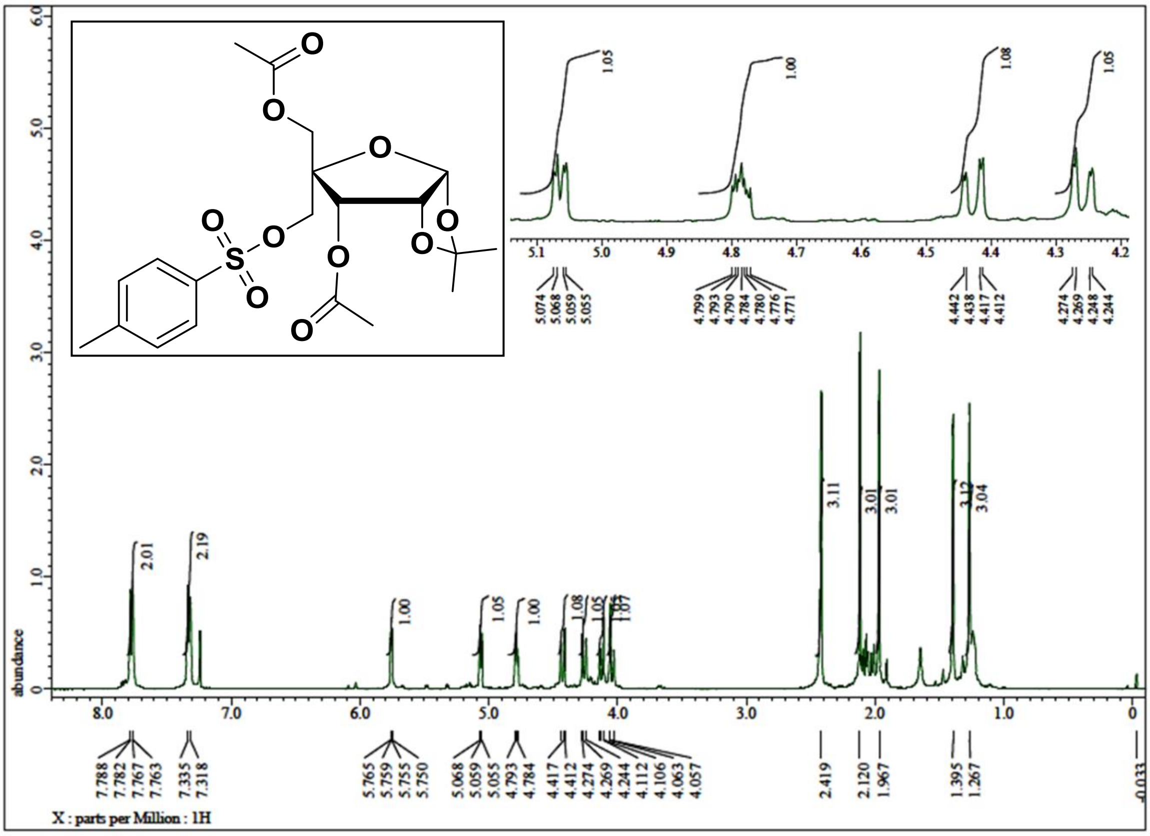

Figure S9: ^{1}H NMR spectrum of compound **6** (400 MHz, $CDCl_3$)

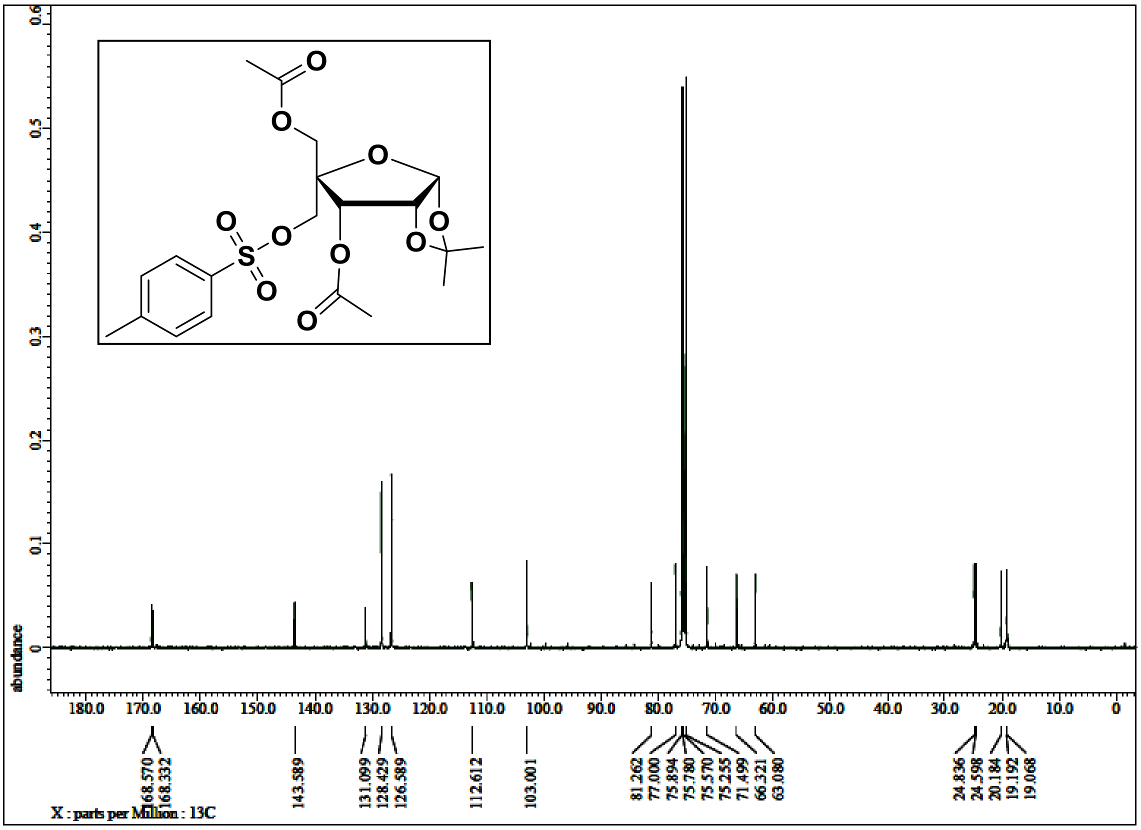

Figure S10: ^{13}C NMR spectrum of compound **6** (100.6 MHz, $CDCl_3$)

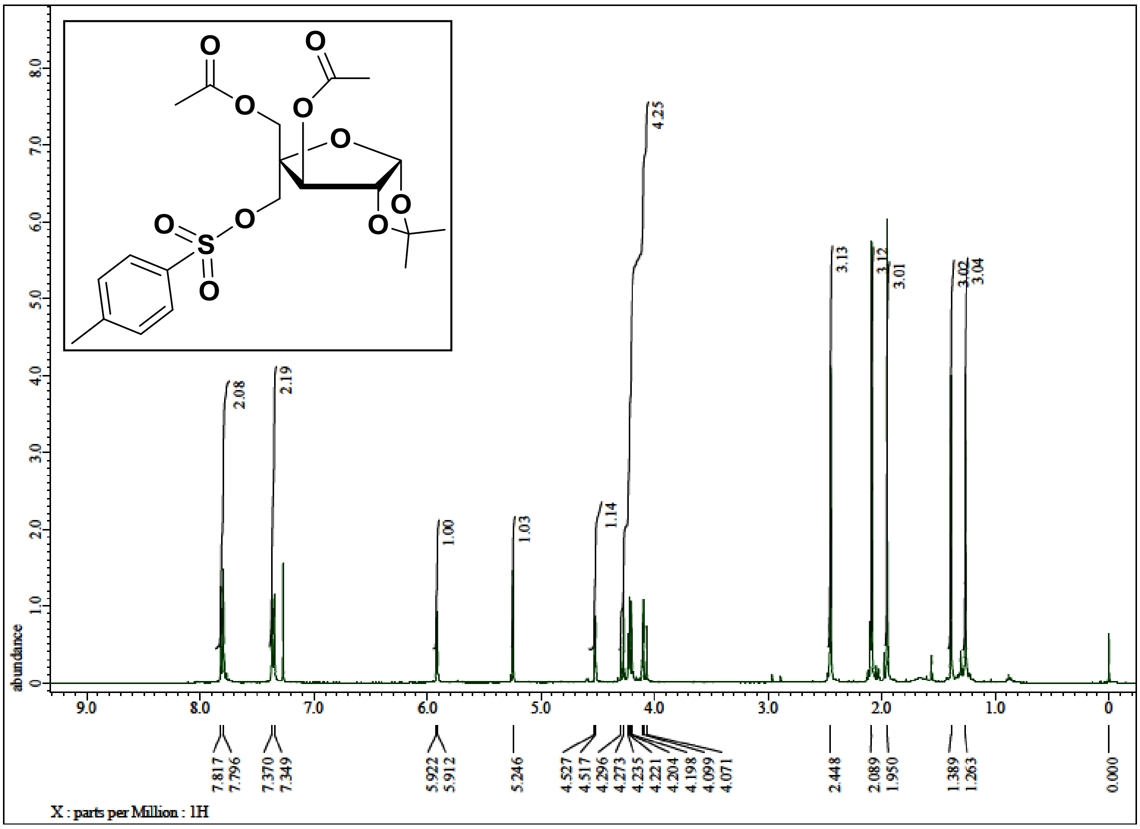

Figure S11: ^{1}H NMR spectrum of compound **11** (400 MHz, $CDCl_3$)

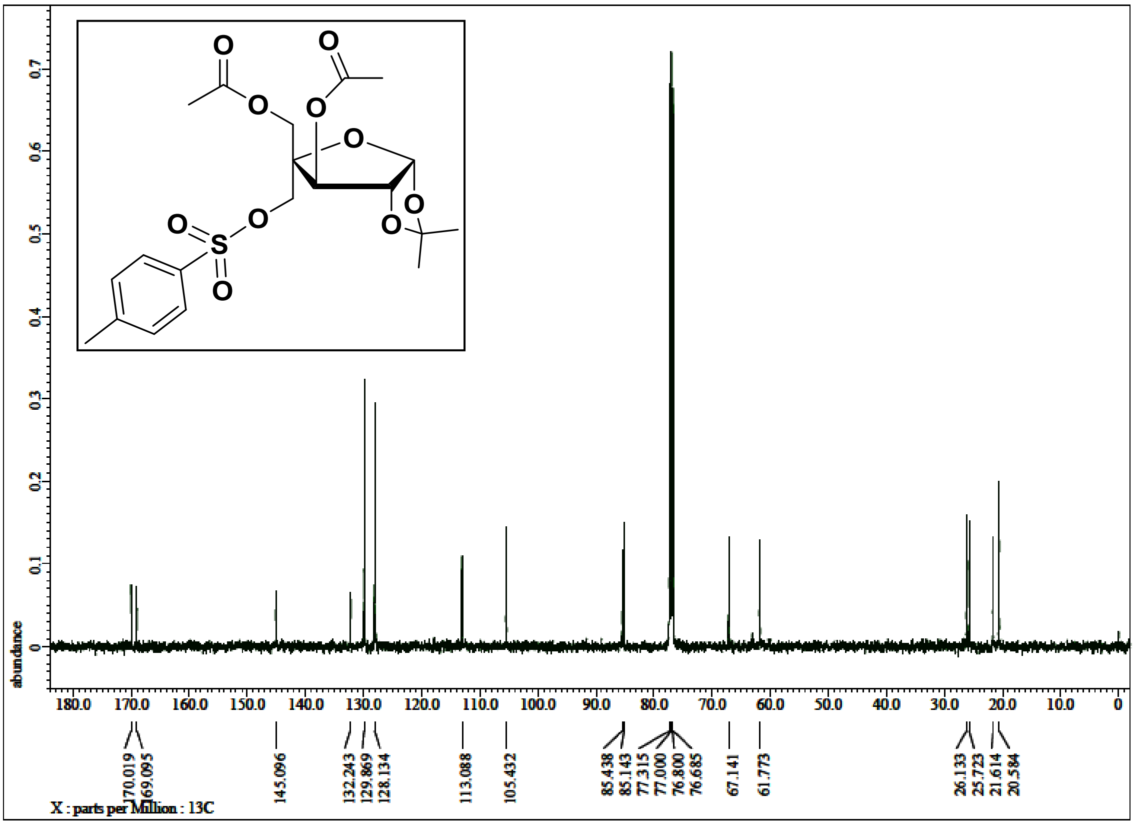

Figure S12: ^{13}C NMR spectrum of compound **11** (100.6 MHz, $CDCl_3$)

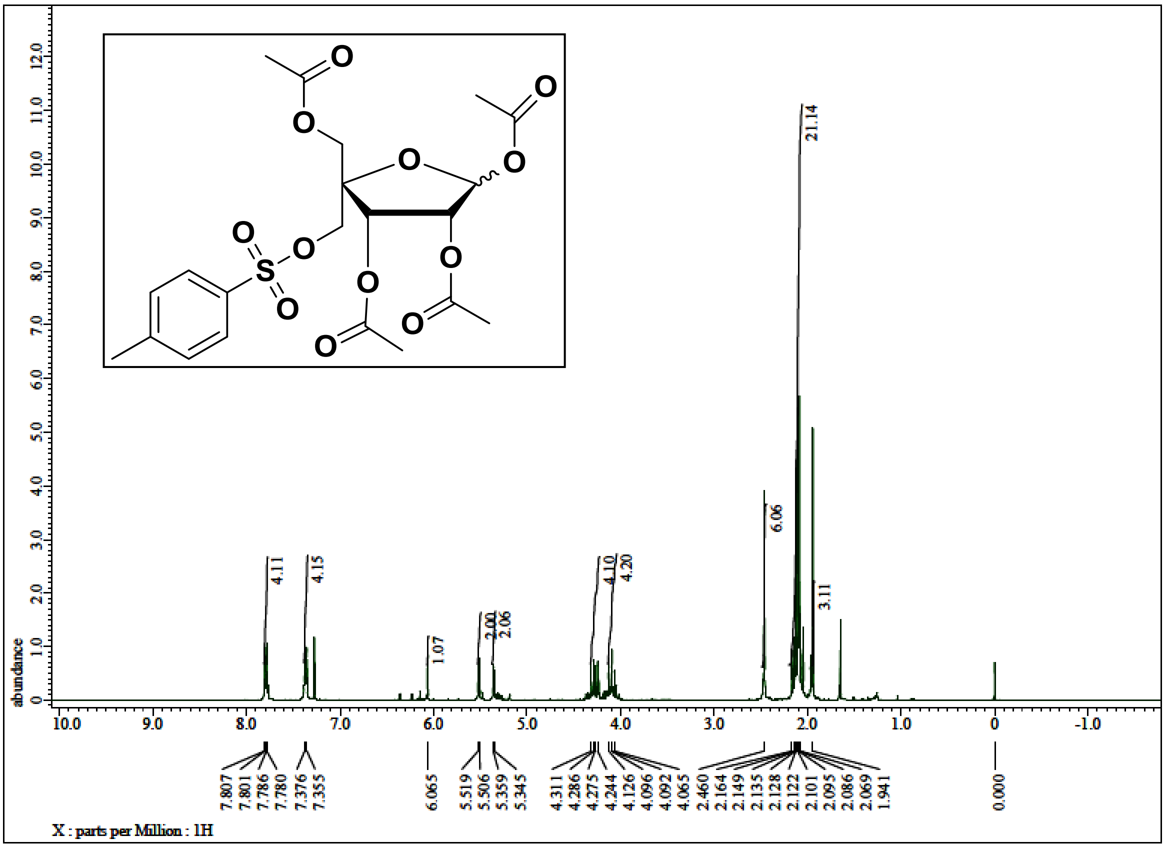

Figure S13: ^{1}H NMR spectrum of compound **7a,b** (400 MHz, $CDCl_3$)

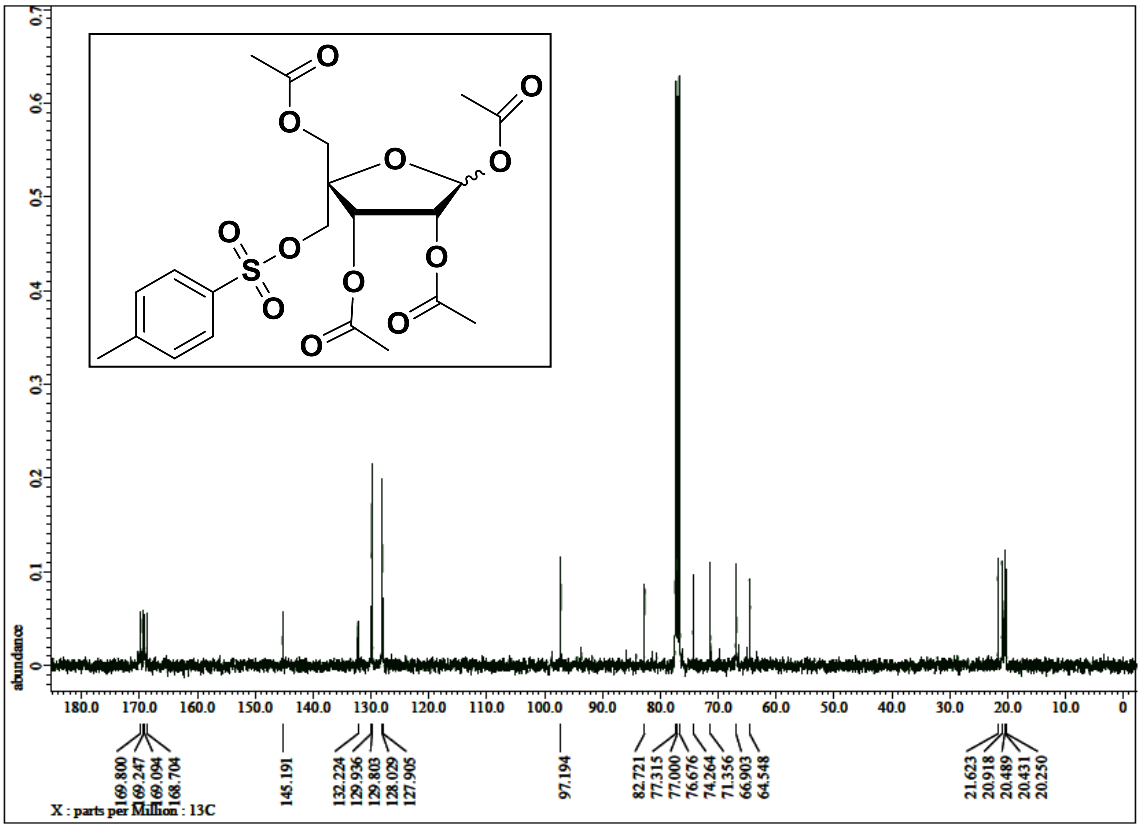

Figure S14: ^{13}C NMR spectrum of compound **7a,b** (100.6 MHz, $CDCl_3$)

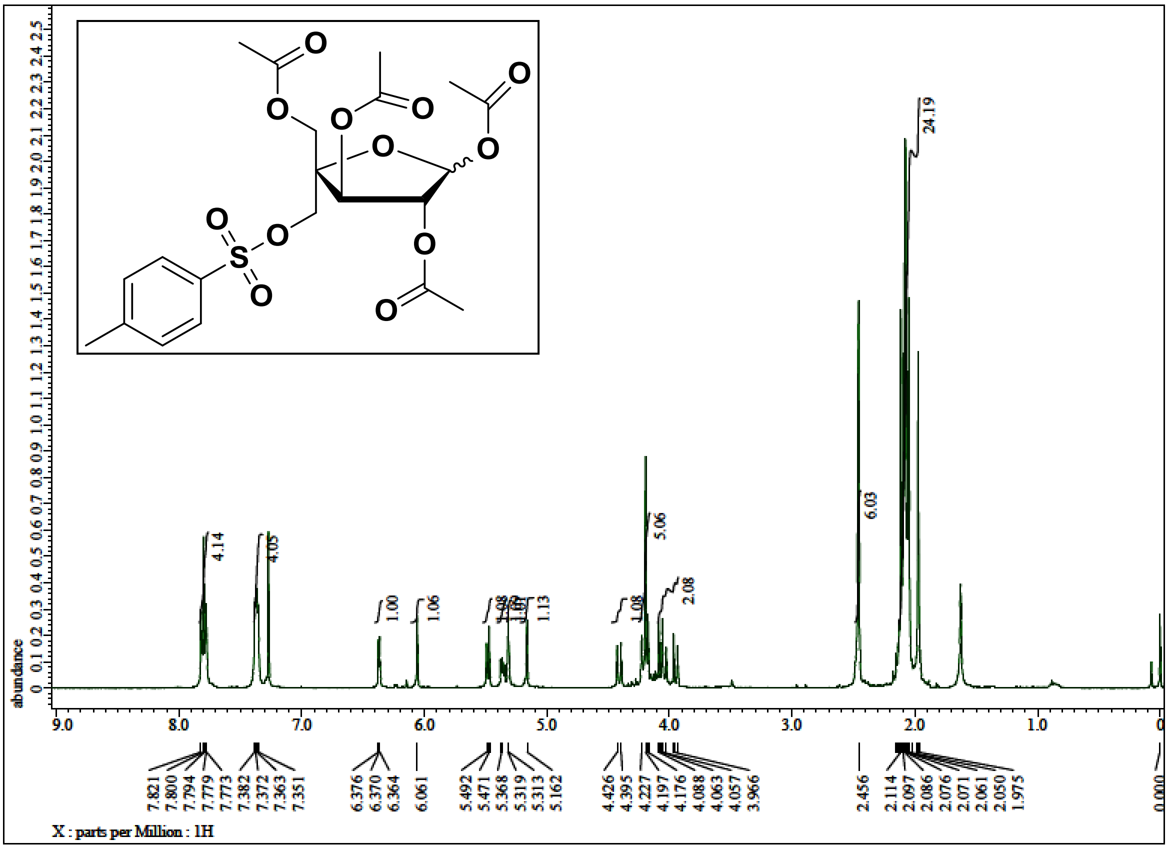

Figure S15: ^{1}H NMR spectrum of compound **12a,b** (400 MHz, $CDCl_3$)

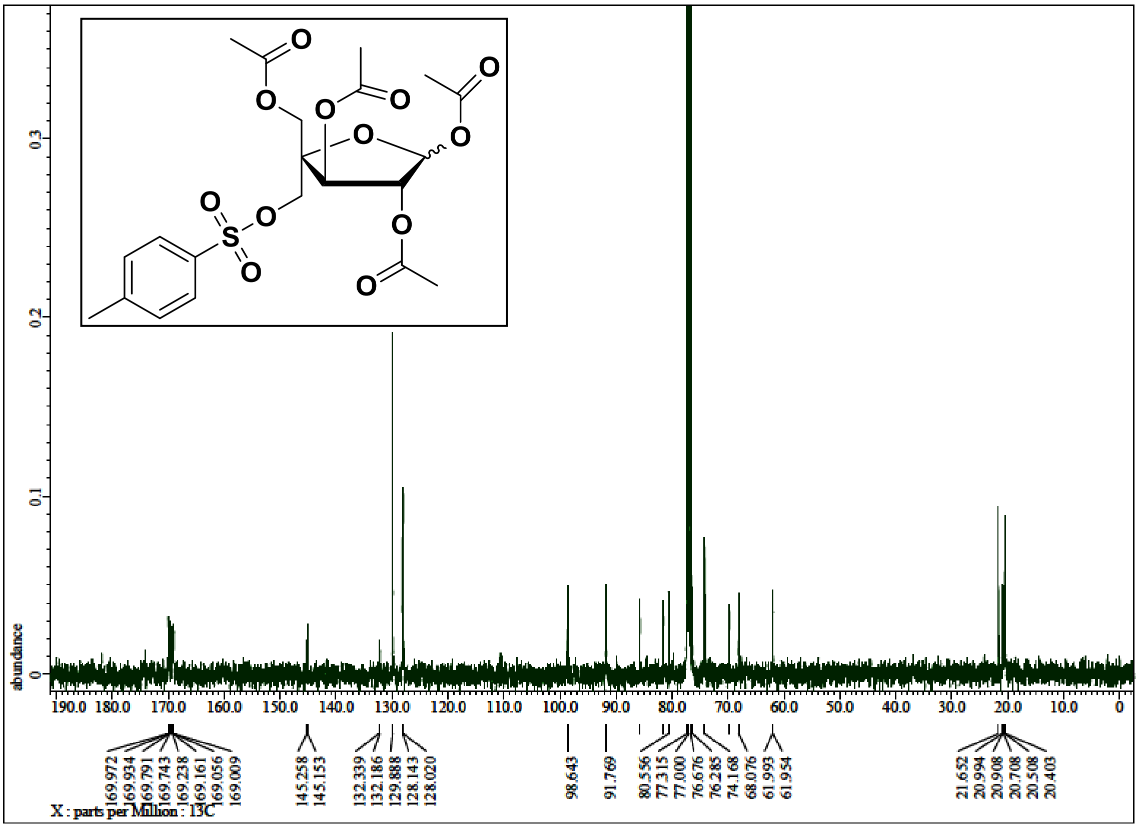

Figure S16: ^{13}C NMR spectrum of compound **12a,b** (100.6 MHz, $CDCl_3$)

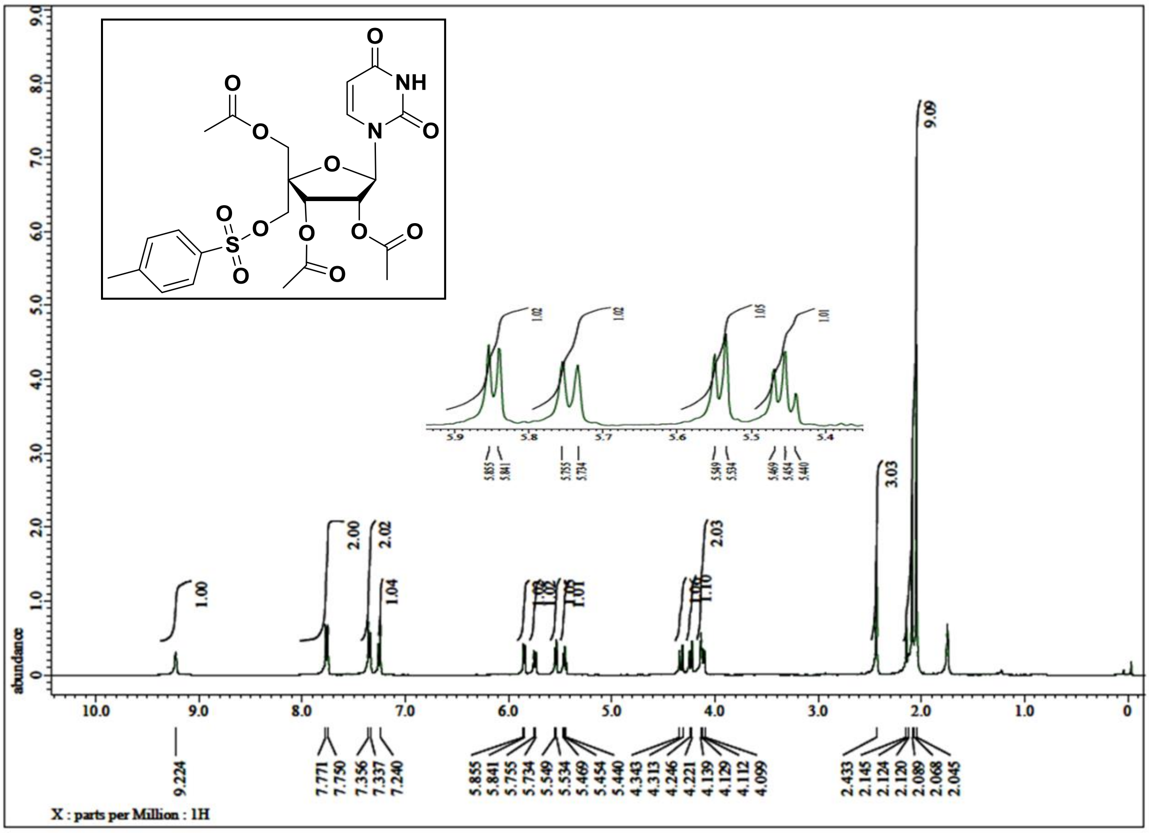

Figure S17: ^{1}H NMR spectrum of compound **8** (400 MHz, $CDCl_3$)

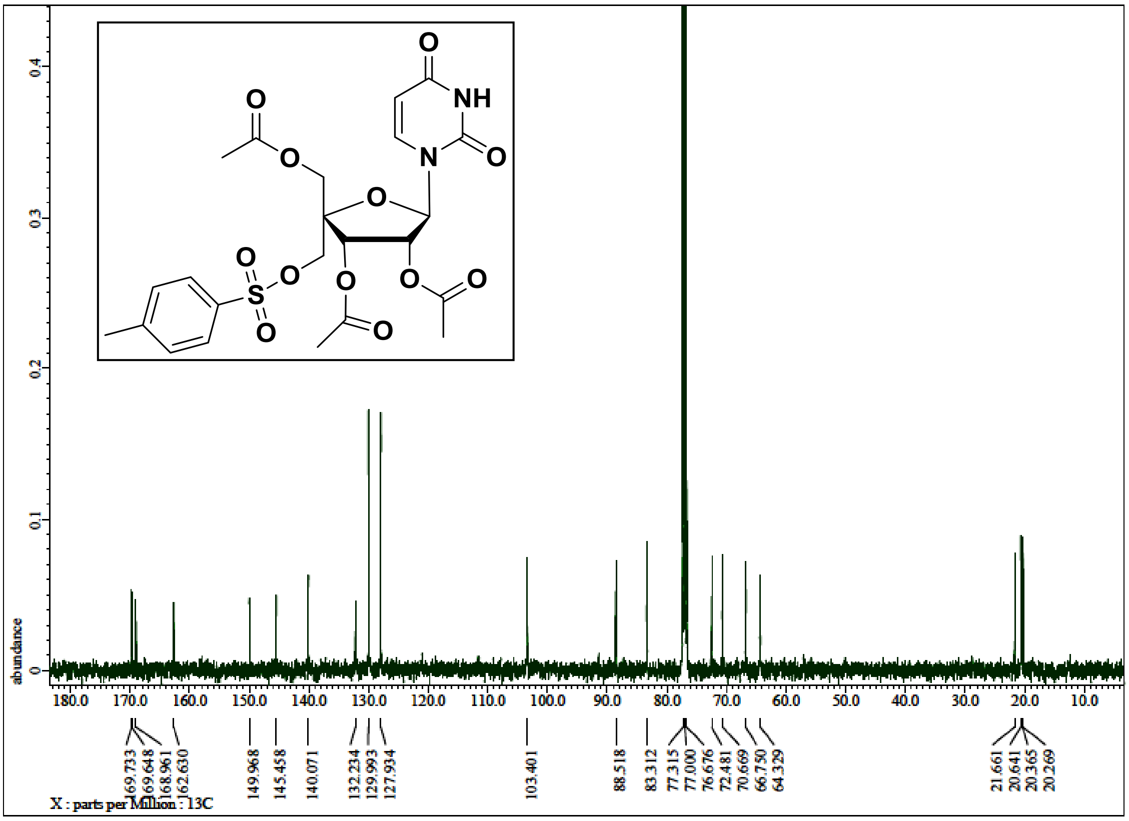

Figure S18: ^{13}C NMR spectrum of compound **8** (100.6 MHz, $CDCl_3$)

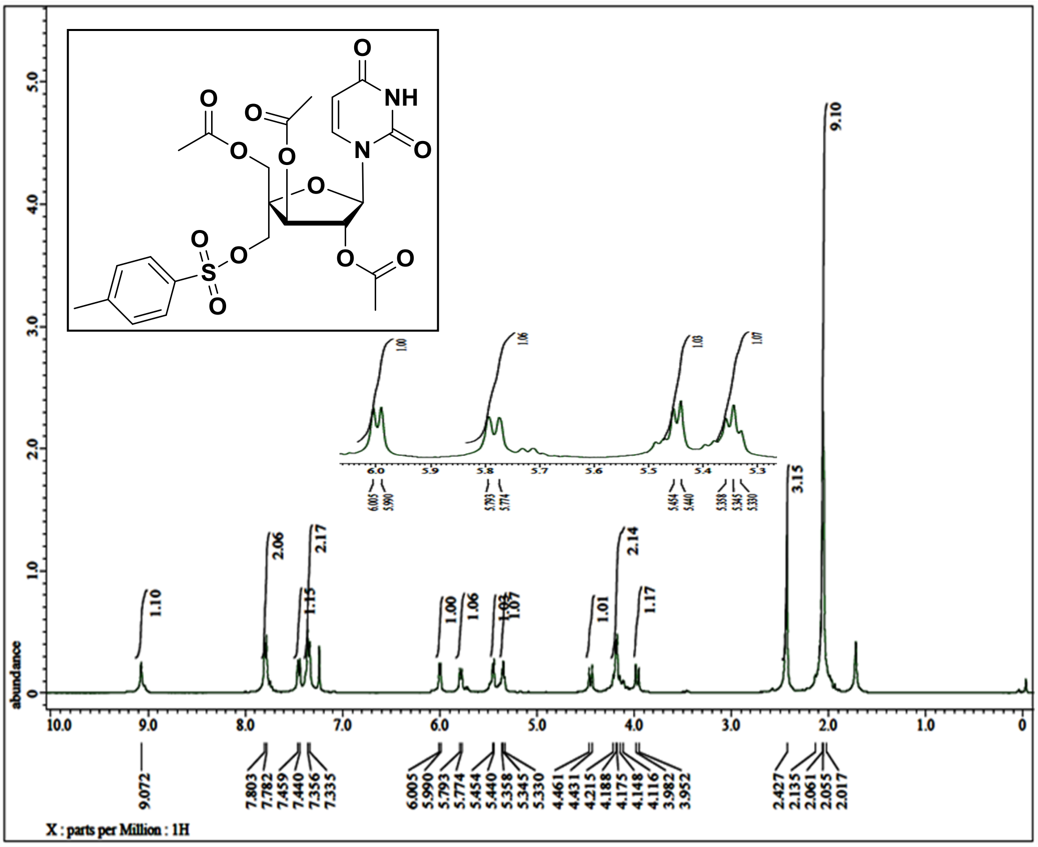

Figure S19: ^{1}H NMR spectrum of compound **13** (400 MHz, $CDCl_3$)

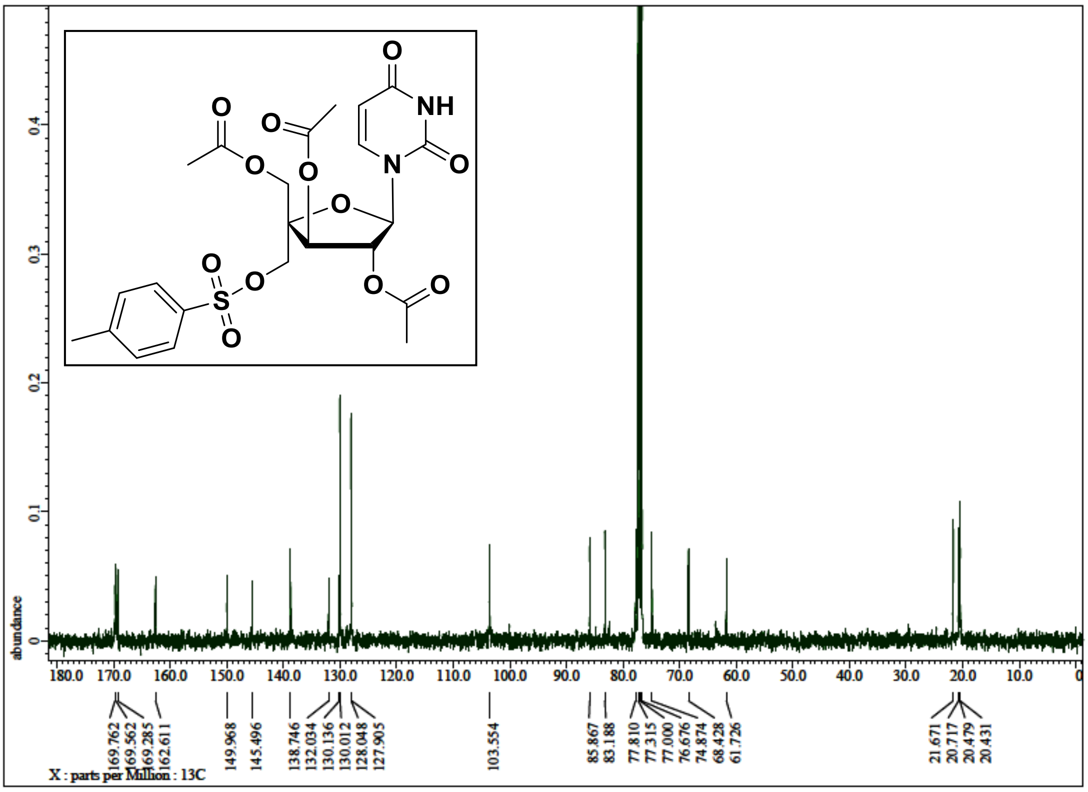

Figure S20: ^{13}C NMR spectrum of compound **13** (100.6 MHz, $CDCl_3$)

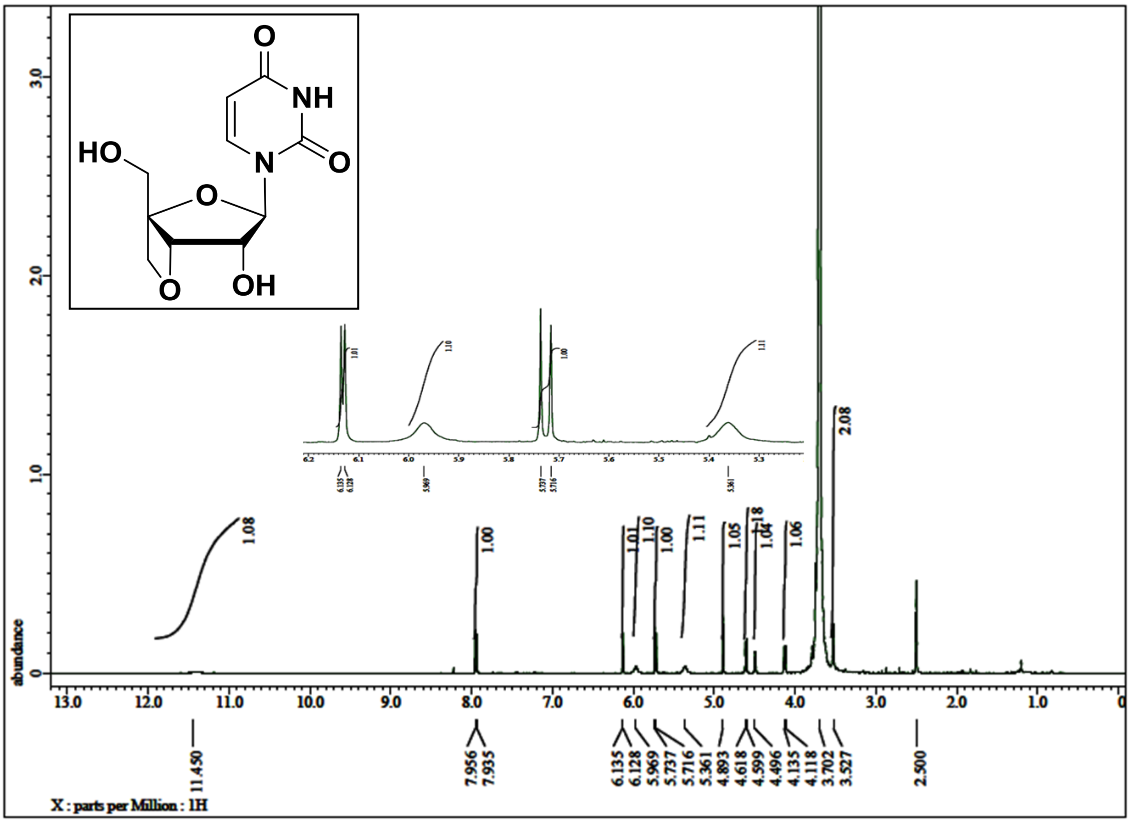

Figure S21: ^{1}H NMR spectrum of compound **9** (400 MHz, DMSO-d_6)

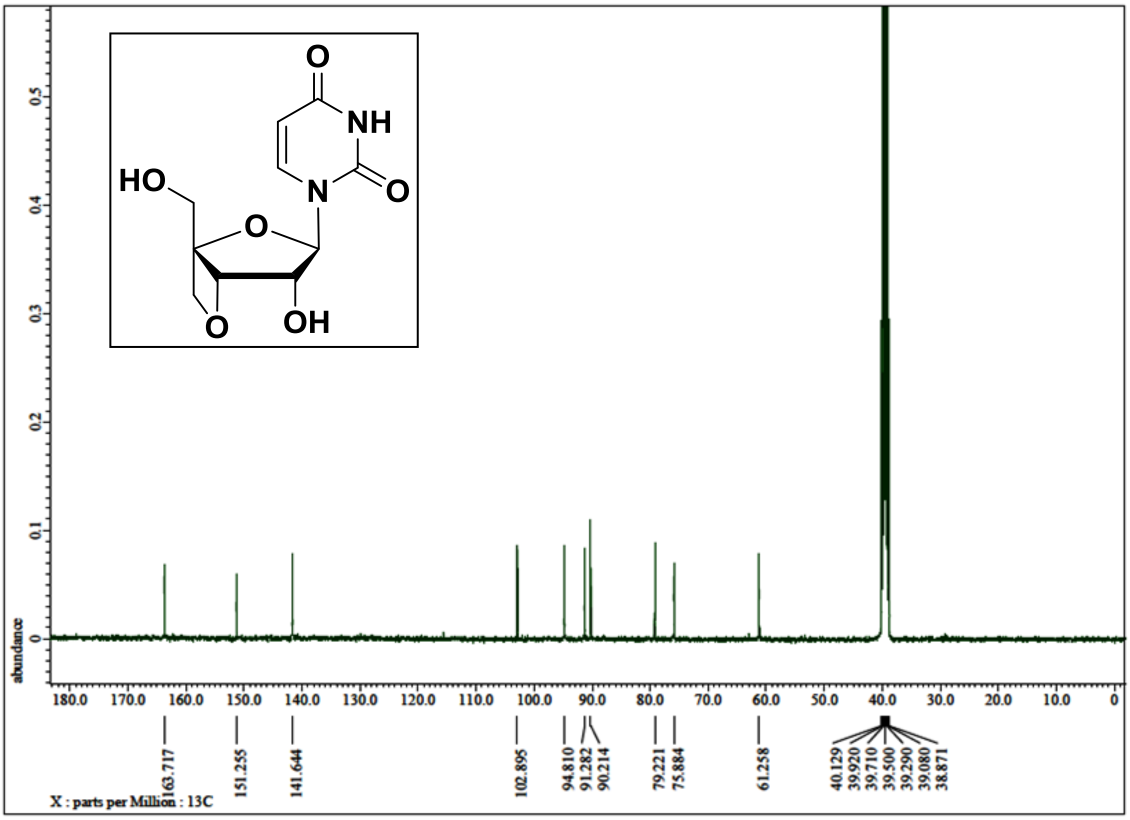

Figure S22: ^{13}C NMR spectrum of compound **9** (100.6 MHz, DMSO-d_6)

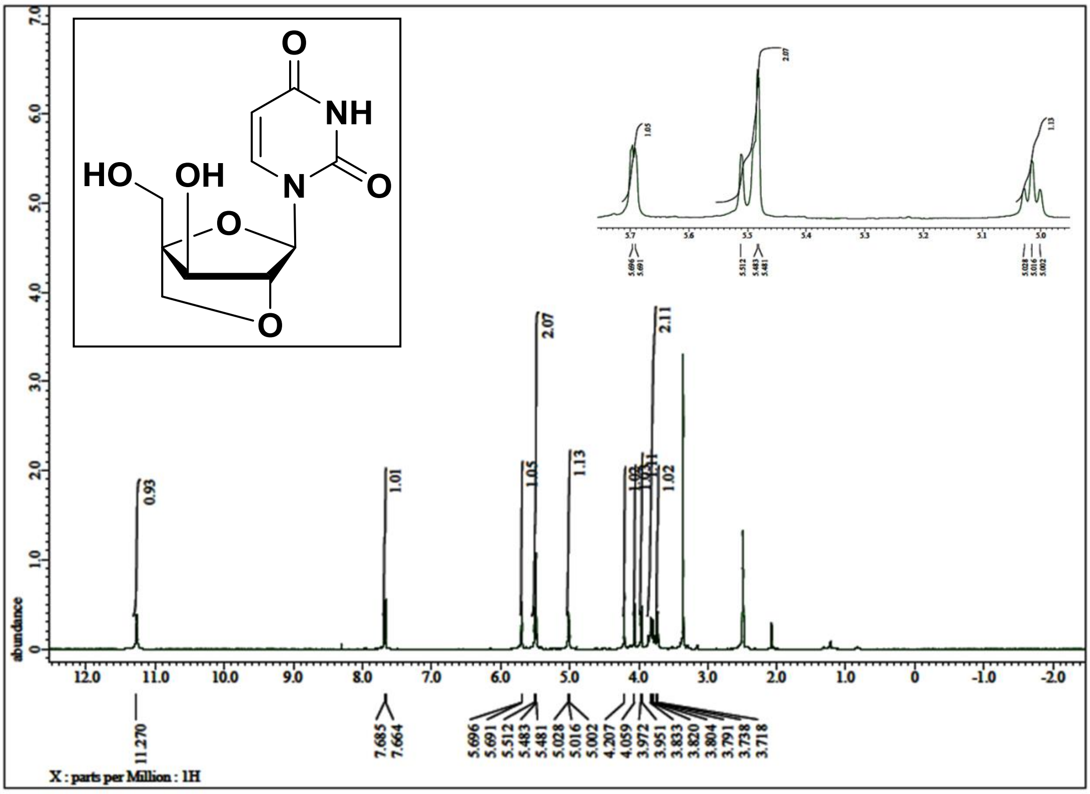

Figure S23: ^{1}H NMR spectrum of compound **14** (400 MHz, DMSO-d_6)

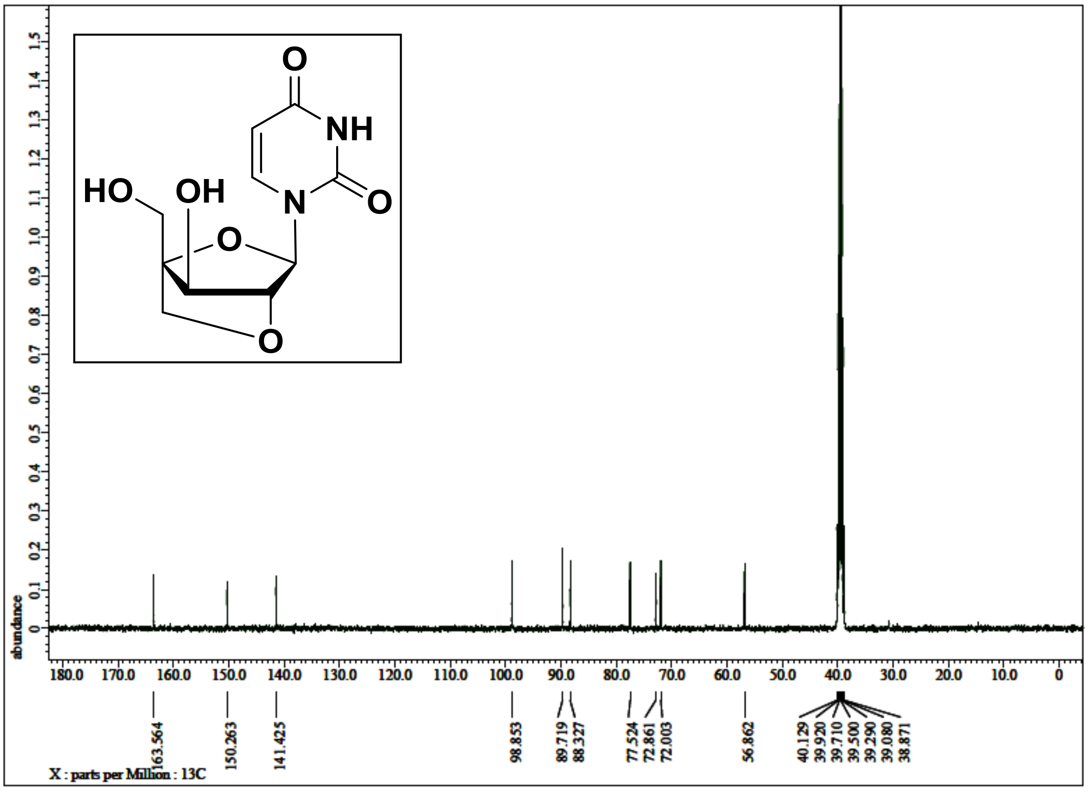

Figure S24: ^{13}C NMR spectrum of compound **14** (100.6 MHz, DMSO-d_6)
